# A numerical population density technique for N-dimensional neuron models

**DOI:** 10.3389/fninf.2022.883796

**Published:** 2022-07-22

**Authors:** Hugh Osborne, Marc de Kamps

**Affiliations:** ^1^School of Computing, University of Leeds, Leeds, United Kingdom; ^2^Leeds Institute for Data Analytics, University of Leeds, Leeds, United Kingdom; ^3^The Alan Turing Institute, London, United Kingdom

**Keywords:** simulator, neural population, population density, software, Python, dynamical systems, network, visualization

## Abstract

Population density techniques can be used to simulate the behavior of a population of neurons which adhere to a common underlying neuron model. They have previously been used for analyzing models of orientation tuning and decision making tasks. They produce a fully deterministic solution to neural simulations which often involve a non-deterministic or noise component. Until now, numerical population density techniques have been limited to only one- and two-dimensional models. For the first time, we demonstrate a method to take an N-dimensional underlying neuron model and simulate the behavior of a population. The technique enables so-called graceful degradation of the dynamics allowing a balance between accuracy and simulation speed while maintaining important behavioral features such as rate curves and bifurcations. It is an extension of the numerical population density technique implemented in the MIIND software framework that simulates networks of populations of neurons. Here, we describe the extension to N dimensions and simulate populations of leaky integrate-and-fire neurons with excitatory and inhibitory synaptic conductances then demonstrate the effect of degrading the accuracy on the solution. We also simulate two separate populations in an E-I configuration to demonstrate the technique's ability to capture complex behaviors of interacting populations. Finally, we simulate a population of four-dimensional Hodgkin-Huxley neurons under the influence of noise. Though the MIIND software has been used only for neural modeling up to this point, the technique can be used to simulate the behavior of a population of agents adhering to any system of ordinary differential equations under the influence of shot noise. MIIND has been modified to render a visualization of any three of an N-dimensional state space of a population which encourages fast model prototyping and debugging and could prove a useful educational tool for understanding dynamical systems.

## 1. Introduction

A common and intuitive method for simulating the behavior of a population of neurons is to directly simulate each individual neuron and aggregate the results (Gewaltig and Diesmann, 2007; Yavuz et al., 2016; Knight et al., 2021). At this level of granularity, the population can be heterogeneous in terms of the neuron model used, parameter values, and connections. The state of each neuron, which may consist of one or many more time or spatially dependent variables, is then integrated forward in time. The benefit of this method of simulation is that it provides a great deal of control over the simulated neurons with the fewest approximations. If required, the state history of each neuron can be inspected. However, this degree of detail can produce results that are overly verbose making it difficult to explain observations. While this can be mitigated by carefully limiting the degrees of freedom (for example, keeping all neurons in the population homogeneous, using point neuron models, or having a well-defined connection heuristic), other simulation methods exist that have such assumptions built in and provide additional benefits like increased computation speed, lower memory requirements, or improved ways to present and interpret the data. For example, so-called neural mass models (Wilson and Cowan, 1972; Jansen and Rit, 1995) eschew the behavior of the individual neurons in a population in favor of a direct definition of the average behavior. These methods are computationally cheap and can be based on empirical measurements but they lack a direct link to the microscopic behavior of the constituent neurons which limits a generalization to populations of different neuron types.

**Figure 1:**
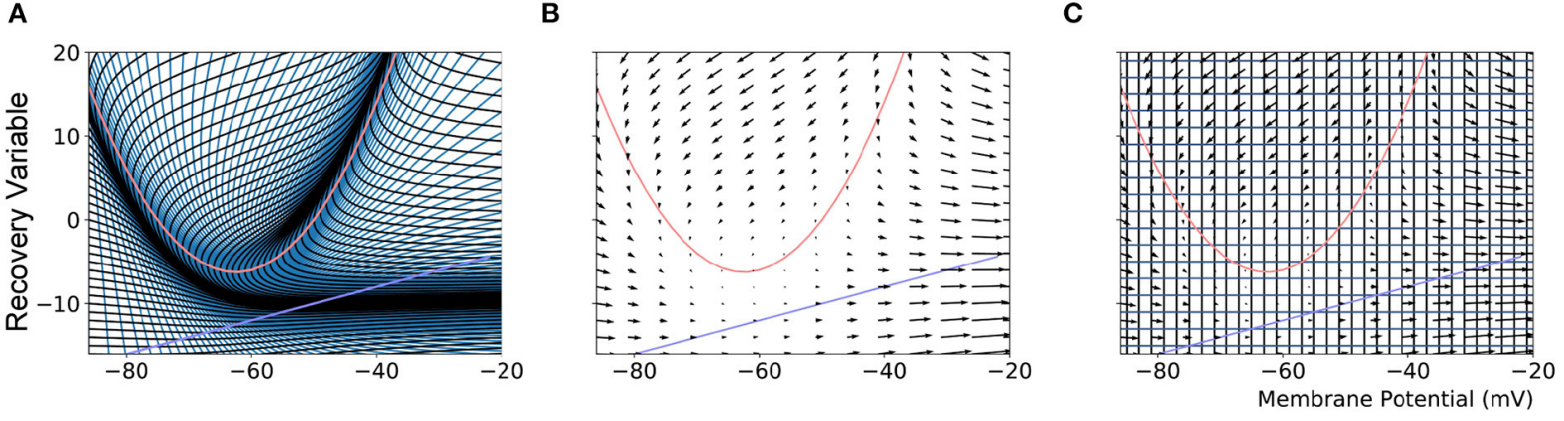
**(A)** The mesh used in MIIND to simulate a population of Izhikevich neurons. The quadratic red curve and blue line are the nullclines where the rate of change of the membrane potential and recovery variable, respectively, are zero. The strips, made up of quadrilateral cells are formed by the characteristic curves of the Izhikevich model for a given parameter set. **(B)** A vector field for the same model showing the direction of movement of probability mass around the state space. **(C)** The state space discretized into a regular grid. The parameters and definition of the Izhikevich model are not given here as it is only required to demonstrate the mesh and grid discretization. As in the original derivation of the model, the recovery variable has no units.

Population density techniques (PDTs) approximate population-level behaviors based on a model definition of the constituent neurons. Most PDTs assume all neurons are homogeneous and unconnected within a discrete population. All neurons are considered point-neurons and adhere to a single neuron model which is made up of one or more variables that describe the state of the neuron at a given time. The state space of the model, as shown in Figure 1, contains all possible states that a neuron in the population could take. For a population of neurons, PDTs frequently define a probability density function or the related probability mass function across the state space which gives the probability of finding a neuron from the population with a given state. PDTs are not concerned with the individual neurons but instead calculate the change to the probability mass function which is governed by two processes: the deterministic dynamics defined by the underlying neuron model, and a non-deterministic noise process representing random incoming spike events.

Methods for solving the deterministic dynamics of a system of ordinary differential equations under the influence of a non-deterministic noise process have been used right back to early studies of Brownian motion. Then in theoretical neuroscience, Johannesma (1969) and Knight (1972) among others used similar techniques to give a formal definition of the effect of stochastic spiking events on a neuron by defining a probability density function of possible somatic membrane potentials. Most often, these involved the assumption of infinitesimal changes in state due to the incoming events, also known as the diffusion approximation. Omurtag et al. (2000) applied the method to a population of unconnected homogeneous neurons. They separated the deterministic dynamics of a common underlying neuron model from the incoming spike train generated by a Poisson process. Originally, the motivation for their work was to more efficiently approximate the behavior of collections of neurons in the visual cortex. Work by Sirovich et al. (1996) showed that there is a lot of redundancy in optical processing in the macaque visual cortex such that on the order of *O*(10^4^) functional visual characteristics or modalities are encoded by *O*(10^8^) neurons. It was, therefore, a reasonable approximation to treat a population of 10^4^ neurons as a homogeneous group and investigate the interaction between populations. The technique was employed by Nykamp and Tranchina (2000) to analyse mechanisms for orientation tuning. Bogacz et al. (2006) also used PDTs to model decision making in a forced choice task.

PDTs have since been extended to attend to various shortcomings of the original formulation. For example, there is often an assumption of Poisson distributed input to a population (Omurtag et al., 2000; Mattia and Del Giudice, 2002; Rangan and Cai, 2007) which in certain circumstances is not biologically realistic. Ly and Tranchina (2009) outlined a technique to calculate the distribution of the output spike train of a population of LIF neurons with different input distributions (based on a renewal process - with a function involving the inter-spike interval). Instead of introducing a Poisson process for their noise term, they use a hazard function which defines the probability of an incoming spike given the time since the last spike. This allows them to handle more realistic input distributions such as a gamma distribution for certain situations and calculate the output firing rate. They are also able to derive the output statistics of a population like expected inter-spike interval and spike distribution. Further work has been done to develop so-called quasi-renewal processes (Naud and Gerstner, 2012) which define the probability of the next spike in terms of both the population level activity and the time since the last spike. Such approaches can simulate behaviors such as spike frequency adaptation and refractoriness but there is a weaker link to the underlying neuron model which limits the simulation of populations of neurons with dynamics that produce behaviors like bursting.

PDTs have also often been limited to low-dimensional neuron models with which to derive population level behavior and statistics. The conductance based refractory density (CBRD) approach (Chizhov and Graham, 2007) tracks the distribution of a population of neurons according to the time since they last spiked (often referred to as their age) instead of across the state space of the neuron model. In its most elementary form, the probability density equation, given in terms of time and time since last spike, is dependent on the neuronal dynamics defined by the underlying model and a noise process. Crucially though, the conductance variables defined in the underlying model (such as the sodium gating variables of the Hodgkin-Huxley neuron model) can be approximated to their mean across all neurons with similar age. With this approximation, the dimensionality of the problem is reduced to a dependence only on the membrane potential, significantly improving the tractability of such systems. Refractory density approaches (Schwalger and Chizhov, 2019) have been extended further to approximate finite size populations, phenomenological definitions, and bursting behaviors (Schwalger et al., 2017; Chizhov et al., 2019; Schmutz et al., 2020).

Using these techniques for modeling and simulation generally requires a large amount of mathematical and theoretical work to develop a solution for a specific scenario. As we see above, each additional behavior requires at least an extension or even reformulation of a previous approach. The numerical PDT implemented in MIIND (de Kamps et al., 2019; Osborne et al., 2021) requires only a definition of the underlying neuron model plus population and simulation parameters. The definition can be given in the form of a Python function in a similar fashion to direct simulation techniques. However, until now, the PDT has been able to simulate populations of neurons adhering to only a one- or two-dimensional model. Often, this is enough as many different neuronal behaviors can be captured with two variables, for example, the action potential of the Fitzhugh-Nagumo neuron (FitzHugh, 1961; Nagumo et al., 1962), the spike frequency adaptation of the adaptive exponential integrate-and-fire neuron (Brette and Gerstner, 2005), or the bursting behavior of the Izhikevich neuron model (Izhikevich, 2007). Using a one-dimensional neuron model, MIIND has been employed to simulate a network of interacting populations in the spinal cord (York et al., 2022). Populations were based on the exponential integrate-and-fire neuron model and showed how a relatively simple spinal network could explain observed trends in a static leg experiment. The main benefit of using the numerical PDT in this study was to eliminate finite-size variation in the results which would have hindered the subsequent analysis. The MIIND software itself also afforded benefits such as the ability to quickly prototype population network models, and to observe the population states during and after simulation. Osborne et al. (2021) have previously presented the full implementation details of MIIND including the two “flavors” of the numerical PDT, named the mesh and grid methods. The mesh method involves discretizing the state space using a mesh of quadrilateral cells as shown in Figure 1A. The grid method was developed chiefly to improve the flexibility of the PDT to avoid building a mesh. In this method, the state space is discretized into a grid of rectangles which allows for a more automated approach. Here, we extend the grid method to greater than two-dimensional models to expand the repertoire of possible neuron types.

## 2. Materials and methods

### 2.1. Recap of the grid method in MIIND

The MIIND algorithm for calculating the change to the probability mass function is covered in detail by de Kamps et al. (2019) and Osborne et al. (2021). However, we will cover the basic algorithm as it is relevant to the extension of the grid method to N dimensions. As a preprocessing step, the state space of the underlying neuron model is discretized such that each discrete volume of state space, or cell, is associated with a probability mass value. The probability mass is assumed to be uniformly distributed across the cell. The discretization can take the form of a mesh as shown in Figure 1A, constructed from the characteristic curves of the underlying neuron model or a regular grid which spans the state space as in Figure 1C.

**Figure 2:**
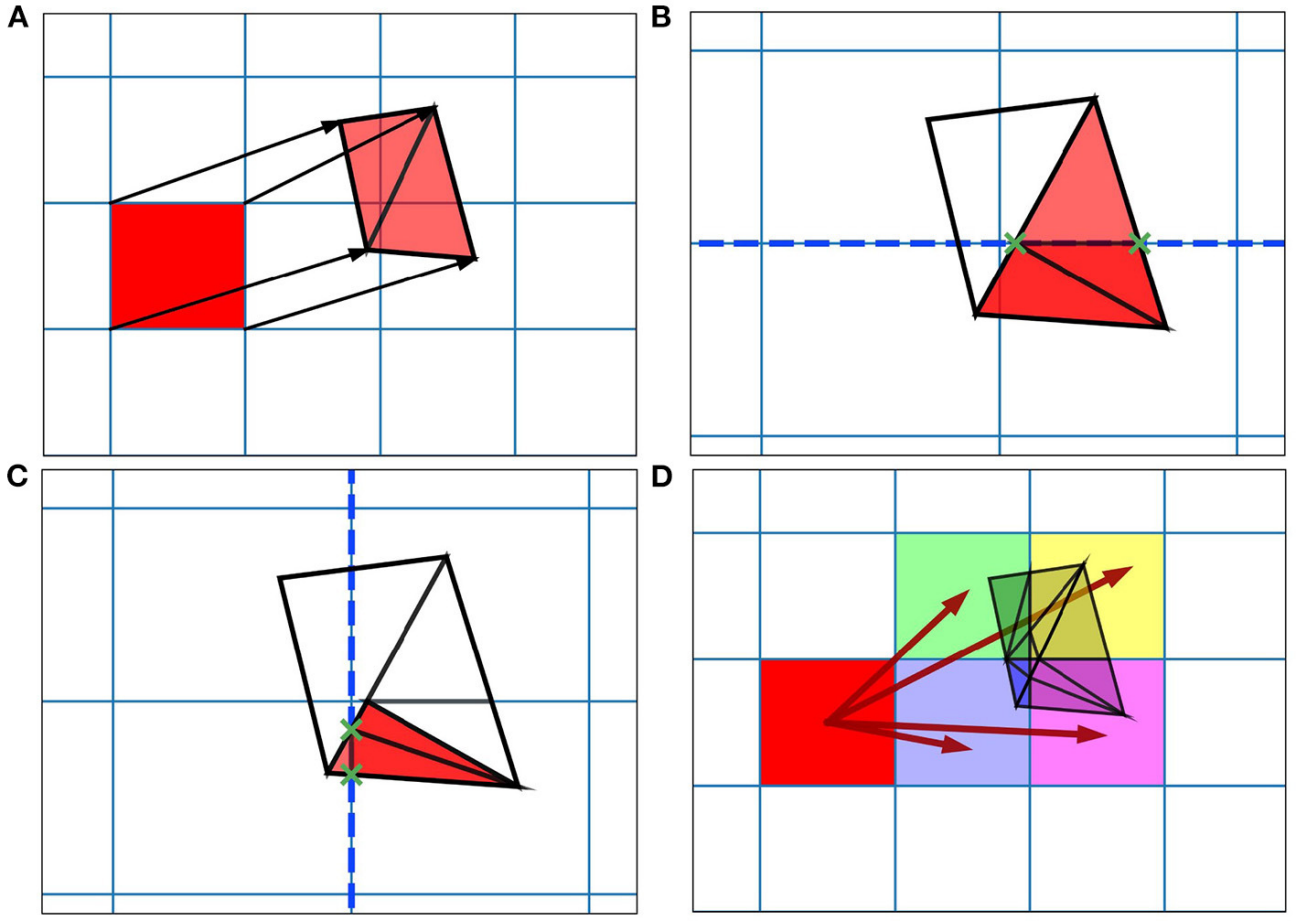
Figure showing steps for generating the transition matrix to solve the deterministic dynamics of the underlying model using a two-dimensional grid. Axes are not labeled as they represent arbitrary time-dependent variables. **(A)** For each grid cell (rectangle), the vertices translated according to a single time step of the underlying neuron model and the resulting quadrilateral is triangulated. **(B)** Each triangle is then tested for intersection with the axis-aligned lines of the original grid. The green crosses mark the intersection points between the tested triangle and the dashed line. The resulting subsections are again triangulated. **(C)** The process runs recursively until no more triangulations can be made. **(D)** The resulting triangles each lie within only a single cell of the original grid. The area of each triangle divided by the area of the original quadrilateral gives a proportion of mass to be transferred from the grid cell to the containing cell. From these, the proportions to be transferred can be summed and the totals stored in the file.

When generating the grid in MIIND, the user provides the resolution of the grid and the size and location in state space within which the population is expected to remain during simulation. For each iteration of the simulation, the distribution of probability mass across the cells is updated, firstly, according to the deterministic dynamics of the underlying neuron model. For example, in the Izhikevich neuron model (Izhikevich, 2007), as shown in Figure 1B, the vector field below −60 mV indicates that probability mass will move slowly toward −60 mV before quickly accelerating to the right. Because the underlying neuron model does not change, the proportion of probability mass transitioning from each cell according to the deterministic dynamics remains constant throughout any simulation and can therefore be precalculated and stored in a file. To generate the file, the steps illustrated in Figure 2 are performed. For each cell, the aim is to calculate where probability mass will move after one time step of the simulation and how much of the mass is apportioned to each cell. First, the four vertices of the cell are translated according to a single time step of the underlying neuron model to produce a quadrilateral which is assumed to remain convex due to the small distance traveled. The quadrilateral is then split into two triangles and each triangle is then processed separately. Each triangle is tested against the axis-aligned edges of the grid. Because the lines are axis-aligned, this is a trivial test for points on either side of the line. If an intersection occurs, the new vertices are calculated to produce two polygons on either side of the line. Each polygon is triangulated and the process is recursively repeated on all sub-triangles until no more intersections occur. Once all triangles have been tested, the quadrilateral is now split into a collection of triangles which are each entirely contained within one cell of the grid. For each cell which contains one or more triangles, the total area of the triangles is calculated as a proportion of the area of the quadrilateral and this value represents the proportion of probability mass which will be transferred from the originating grid cell after one time step. It is expected that each transformed cell will only overlap with a few others in the grid so that an *N* × *N* matrix of transitions where *N* is the number of cells should be sparsely populated and can be stored in a file then read into memory. The transitions in the file are applied once every iteration of the simulation. This is a computationally time efficient way to solve the deterministic dynamics.

**Figure 3:**
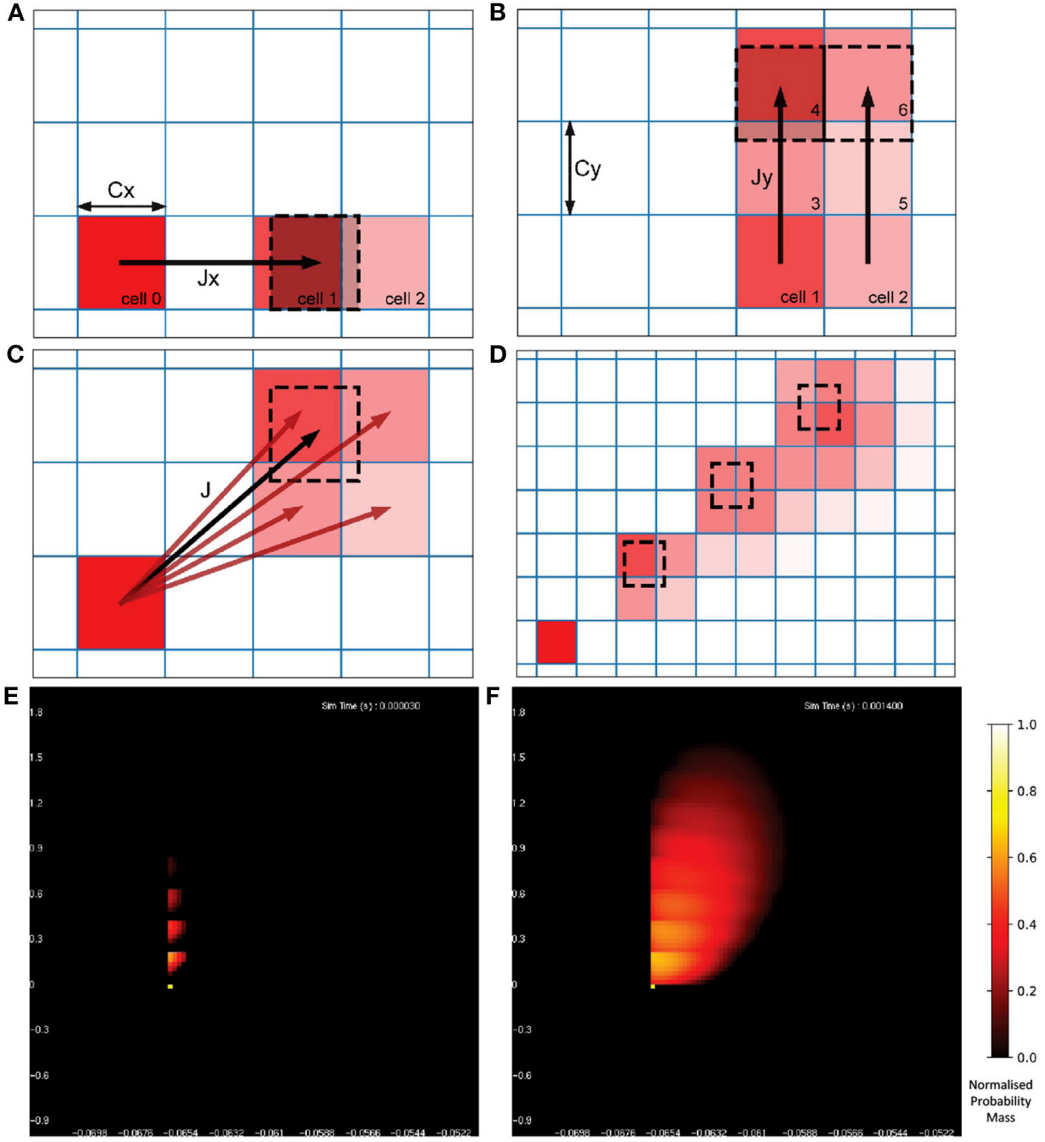
**(A)** The change in state, J, of a neuron due to a single incoming spike can be split into component parts, Jx and Jy for the horizontal and vertical dimensions, respectively. All neurons with a state within cell 0 will be translated by Jx due to a single incoming spike. Because all cells are the same width (Cx), the uniformly distributed probability mass of cell 0 will be shared among a maximum of two cells, cell 1 and cell 2. The offset of cell 1 from cell 0 is equal to *floor*(*Jx*/*Cx*) [for negative Jx, it is *ceil*(*Jx*/*Cx*)] with cell 2 being the one beyond that. The proportion of mass transferred from cell 0 to cell 1 is equal to 1 − (*Cx*%*Jx*) and the remainder is transferred to cell 2. **(B)** Once the mass proportions have been calculated in the horizontal direction, the same calculations are made with cells 1 and 2 in the vertical direction using Cy and Jy. The proportion calculated from cell 0 to cell 1 is split between cells 3 and 4. The proportion in cell 2 goes to 5 and 6. **(C)** The proportions of mass to be transferred from cell 0 to the resulting four cells give an approximation of the effect of transition J. With a constant J, this calculation gives the same relative results for every cell and therefore only needs to be performed once. **(D)** Iteratively applying the transitions to all cells in the grid spreads mass further across state space simulating the effect of neurons receiving multiple spikes in a given time step. **(E)** The probability mass function of a population of leaky integrate-and-fire neurons with an excitatory synaptic conductance rendered in MIIND. The color of each cell indicates the amount of probability mass. The value has been normalized to the maximum value of all cells. The effect of an incoming spike is to shift mass 0.2 nS/cm^2^ in the vertical direction (producing a change in synaptic conductance). At this early point in the simulation, most neurons would have received zero or one spike (indicated by the bright yellow spots) while only a few would have received up to four spikes. **(F)** As the simulation proceeds, mass continues to be transferred upwards due to incoming spikes but the deterministic dynamics of the model causes mass to also move to the right according to the transitions defined in the matrix file and the population becomes more cohesive.

Once the probability mass distribution has changed according to the deterministic dynamics of the underlying neuron model, the second part of the MIIND algorithm calculates the spread of mass across cells due to random (usually Poisson distributed) incoming spikes. This process is more computationally expensive than the first because the shape of the spread must be recalculated every time step by solving the Poisson master equation (de Kamps, 2006), which involves iteratively applying a different set of transitions to the probability mass function and is dependent on the incoming rate of spikes. Figure 3 shows how the spread of probability mass can be calculated in two dimensions based on the width of the cells and the change in state due to a single incoming spike. Calculating the transitions for solving the non-deterministic noise process benefits from the fact that all cells are the same size and regularly spaced. It is assumed that a single incoming spike will cause a neuron's state to instantaneously jump by a constant vector, *J*. Most often this is only in one direction instead of two. For example, many neuron models expect an instantaneous jump in membrane potential or in synaptic conductance. However, calculating the jump transition for any vector is a useful feature to have for models like the Tsodyks-Markram synapse model (Tsodyks and Markram, 1997) for which incoming spikes cause a jump in two variables at once. For a single incoming spike, all probability mass in a cell will shift up or down according to the *x* component of *J*, where *x* is the first variable or dimension of the model. Because all cells are the same size, this shift will result in probability mass being shared among at most two other cells which are adjacent to each other. Calculating which cells receive probability mass and in what proportion requires only knowing the width of the cells in the *x* dimension and the *x* component of *J*. If the *J* vector has a *y* component, where *y* is the second variable or dimension, the same process can be applied to each of the two new cells. The proportion of probability mass to be shared to each cell is itself shared among a further two cells for a maximum of four cells containing probability mass from the original cell. Due to the regularity of the grid, this calculation need only be made once and is applicable to every other cell. To simulate the effect of the incoming Poisson noise process on the probability mass function, the transitions are applied iteratively to each cell.

Figures 3E,F show the resulting probability mass function during a simulation when both deterministic and non-deterministic processes are applied. From the function, average values across the population can be calculated as well as the average firing rate if the underlying model has a threshold-reset mechanism. In that case, after each iteration, mass that has moved into the cells that lie across the threshold potential is transferred to cells at the rest potential according to a mapping generated during the pre-processing steps. The details of this mechanism are described by Osborne et al. (2021).

### 2.2. Extending the grid to N dimensions

**Figure 4:**
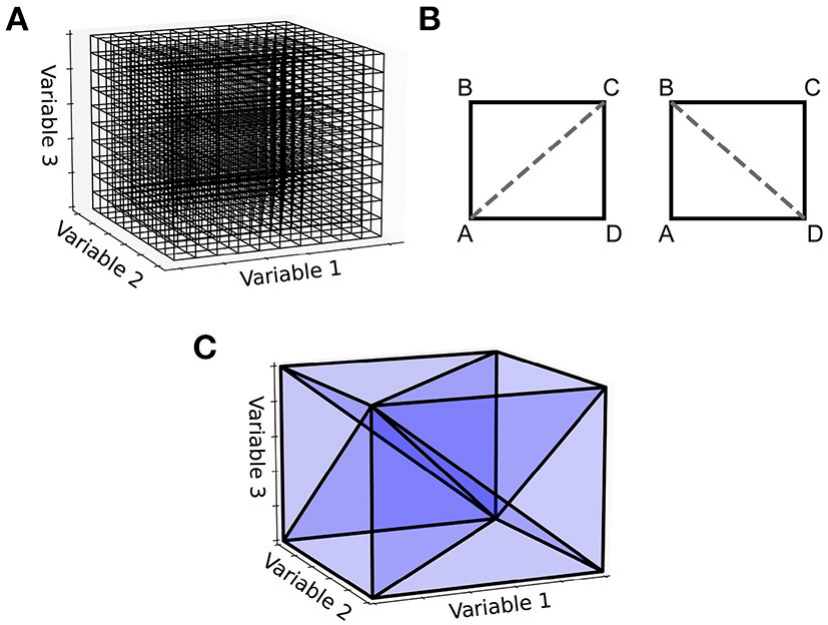
**(A)** With a three-dimensional state space, the grid discretization is made up of cuboids. **(B)** For the two-dimensional case, a rectangle has two possible triangulations, [A,B,C] and [A,C,D] or [A,B,D] and [B,D,C]. **(C)** A cuboid triangulated into six 3-simplices. Other triangulations are possible, some which aim to achieve the minimum number of simplices or to keep the volumes of the simplices as uniform as possible. The Delaunay triangulation makes no guarantees of this kind but is easy to implement and works in N-dimensions.

An important observation is that the steps shown in Figure 2 for generating the two-dimensional transition matrix file work similarly in higher dimensions. However, the complexity of the algorithm increases significantly. For a three-dimensional underlying neuron model, the grid is extended such that each cell is a cuboid in state space with eight vertices (Figure 4). For an N-dimensional (ND) neuron model, an N-dimensional grid can be constructed with cells made up of 2^*N*^ vertices. The task here is to update the calculations involved in the deterministic and non-deterministic processes described above so that they work generically for any number of dimensions. For illustration purposes, we will use a three-dimensional grid.

For the deterministic dynamics, each of the 2^*N*^ vertices is again translated according to a single time step of the neuron model and the resulting volume must be triangulated into N-simplices. In three dimensions, a 3-simplex is a tetrahedron. There are many possible triangulations of an N-dimensional cell. As an example, in the simpler two-dimensional case, if the four vertices of a rectangle are labeled A to D in a clockwise fashion as in Figure 4B, the possible triangulations are [A,B,C] and [A,C,D] or [A,B,D] and [B,D,C]. As with the number of possible triangulations, the number of resulting N-simplices increases with dimensionality and there are many algorithms available to generate them (Haiman, 1991). Many algorithms exist to find the so-called Delaunay triangulation of a set of points, which has a specific definition: A set of triangles (or N-simplices) between points such that no point lies within the circumcircle (or hypersphere) of any triangle (or N-simplex) in the set. This definition results in a quite well-formed triangulation (minimizing the number of long and thin triangles). One of the simplest ways to find the Delaunay triangulation of a set of points in N dimensions is to use the quickhull algorithm (Brown, 1979; Barber et al., 1996). The initial triangulation of the transformed cell is calculated using this method. To improve efficiency of this triangulation step, instead of finding the Delaunay triangulation for every translated cell, quickhull can be applied once to a unit N-cube as shown in Figure 4C. Under the assumption that the transformed cell remains a convex hull (not unreasonable given that the time step should be small), the triangulation of the unit N-cube can be applied to every transformed cell without re-calculating.

**Figure 5:**
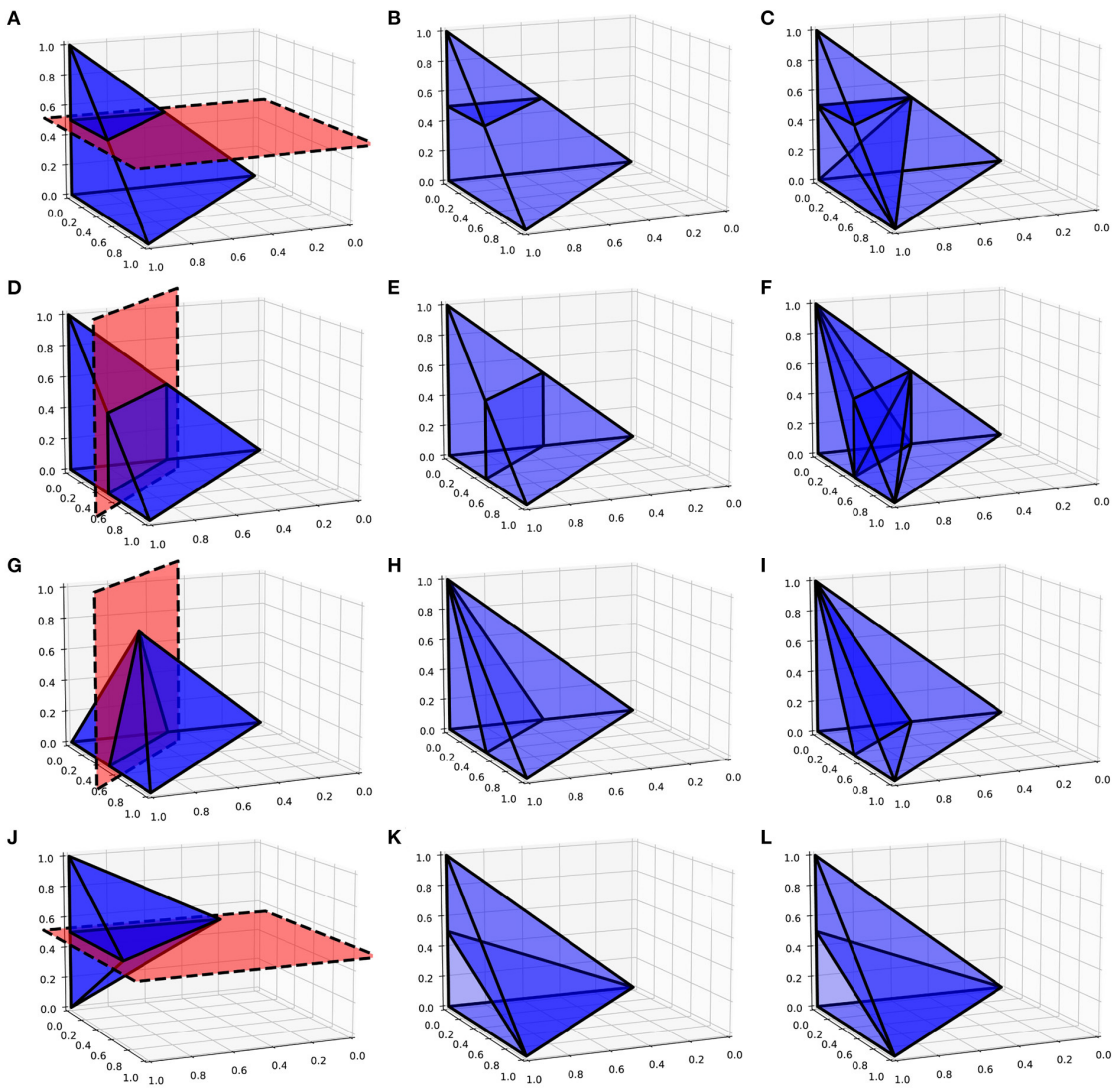
**(A,D,G,J)** Possible plane intersections with a 3-simplex. **(B,E,H,K)** Illustration of how each intersection is represented in the algorithm such that intersections bisect the relevant edges. **(C,F,I,L)** The resulting triangulations of the bisected 3-simplex which can be applied to all intersections of this type when calculating the transitions. **(A–C)** A plane intersection leaving one vertex of the 3-simplex above the plane and three vertices below. **(D–F)** A plane intersection leaving two vertices on either side of the plane. **(G–I)** A plane intersection which goes through one of the vertices leaving one vertex above the plane and two vertices below. **(J–L)** A plane intersection which goes through two vertices leaving one vertex on either side.

As with the two-dimensional version, the next step is to recursively test each N-simplex for intersections with hyperplanes of the grid. Figure 5 shows examples of possible plane intersections of a 3-simplex. Finding an intersection, again, trivially involves checking if vertices lie on both sides of the hyperplane. The new vertices resulting from the intersections with the edges of the N-simplex describe two new shapes on either side of the plane. These must again be triangulated into smaller N-simplices. As with the first triangulation of the unit N-cube, pre-calculated triangulations of a unit N-simplex can be mapped to each newly generated N-simplex of the transformed cell. However, as Figure 5 shows, there are multiple ways that an N-simplex can be bisected with each requiring a different triangulation of the resulting shapes. Each type of intersection can be described uniquely with the number of vertices above the hyperplane, below the hyperplane and on the hyperplane. Table 1 gives the possible bisections of a 3-simplex which are illustrated in Figure 5. The terms “above” and “below” are just used here to describe each side of the hyperplane and do not represent a position relative to each other or the hyperplane. Listing 1 gives the programmatic way to find all possible intersections of an N-simplex.

**Listing 1 Calculate all possible vertex combinations to uniquely identify each type of intersection of an N-simplex**


  For each possible number of co-planar vertices
      which **is** between 0 **and** 2^N - 2:
      List all possible combinations of the
          remaining vertices above **and** below the
          hyperplane excluding 0


**Table 1:** Possible vertex configurations from bisections of a 3-simplex.

**Vertices above the plane**	**Vertices below the plane**	**Vertices on the plane**	**Resulting new vertices**
1	3	0	3
2	2	0	4
1	2	1	2
1	1	2	1

For each of the vertex combinations which uniquely identifies a type of intersection, the appropriate triangulation of the resulting shapes can be pre-calculated using the Delaunay triangulation of a unit N-simplex. To do this, the vertices of the N-simplex are assigned to be “above”, “below” or “on” according to the vertex combination. At this point, no hyperplane exists to test for intersection points. However, we know that edges that pass between an “above” vertex and a “below” vertex will be intersected so we can choose to bisect that edge to produce a new vertex as shown in Figure 5. This represents a good enough approximation of the eventual N-simplex bisection and the quickhull algorithm can be performed on the resulting two shapes. The full dictionary of vertex combinations to triangulations is stored in a lookup table so that, during the actual subdivision of N-simplices in the grid, all that is required is to find the correct intersection in the table and to apply the triangulation mapping. As before, the algorithm continues recursively until no more triangulations are required and the volumes of all N-simplices are summed to calculate the proportion of probability mass which will be shared among the relevant cells.

Solving the non-deterministic dynamics in N dimensions is precisely the same as for two dimensions. In the same way that the probability mass proportion was recursively shared among two new cells per dimension, the resulting number of cells to which mass is transitioned due to a single incoming spike is at most 2^*N*^. No intersections of triangulations are required for this calculation as only the cell width and the jump value in each dimension is required as shown in Figure 3. The MIIND algorithm proceeds in the same way as it did for two dimensions. First applying the matrix of transitions for the deterministic dynamics to the grid, then iteratively applying the jump transition to each cell multiple times to approximate the spread of probability mass due to Poisson distributed input. If the underlying neuron model has a threshold-reset mechanism, probability mass in the cells at threshold (for a three-dimensional grid, this is a two-dimensional set of cells) is transferred to a set of reset cells according to another pre-calculated mapping.

### 2.3. Running an ND simulation in MIIND

When implementing the ND extension to the grid method in MIIND, care has been taken to minimize any changes to how the user builds and runs a simulation. Listing 2 shows a MIIND simulation file for defining two neuron populations in an E-I configuration as examined later in Section 2.5.

**Listing 2 The XML-style simulation file for an E-I network in MIIND**


<Simulation>
<WeightType>CustomConnectionParameters </
    WeightType>
<Algorithms>
<Algorithm type=''GridAlgorithmGroup'' name=''
    COND3D'' modelfile=''cond3d.model''
    tau_refractive=''0.002'' transformfile=''
    cond3d.tmat'' start_v=''0-65'' start_w=''0.00001
    '' start_u=''0.00001''>
<TimeStep>1e-03</TimeStep>
</Algorithm>
</Algorithms>
<Nodes>
<Node algorithm=''COND3D'' name=''E'' type=''
    EXCITATORY'' />
<Node algorithm=''COND3D'' name=''I'' type=''
    INHIBITORY'' />
</Nodes>
<Connections>
<IncomingConnection Node=''E'' num_connections=''10
    '' efficacy=''0.15'' delay=''0.0'' dimension=''1''
    />
<IncomingConnection Node=''I'' num_connections=''10
    '' efficacy=''0.15'' delay=''0.0'' dimension=''1''
    />
<Connection In=''E'' Out=''E'' num_connections=''50''
    efficacy=''1'' delay=''0.003'' dimension=''1''/>
<Connection In=''I'' Out=''E'' num_connections=''50''
    efficacy=''4'' delay=''0.003'' dimension=''2''/>
<Connection In=''E'' Out=''I'' num_connections=''50''
    efficacy=''1'' delay=''0.003'' dimension=''1''/>
<Connection In=''I'' Out=''I'' num_connections=''50''
    efficacy=''4'' delay=''0.003'' dimension=''2''/>
</Connections>
<Reporting>
    <Display node=''E'' />
   <Average node=''E'' t_interval=''0.001'' />
   <Average node=''I'' t_interval=''0.001'' />
   <Rate node=''E'' t_interval=''0.001'' />
   <Rate node=''I'' t_interval=''0.001'' />
</Reporting>
<SimulationRunParameter>
<master_steps>10</master_steps>
<t_end>TE</t_end>
<t_step>1e-03</t_step>
<name_log>cond.log</name_log>
</SimulationRunParameter>
</Simulation>


The full details of the syntax for a simulation file is provided by Osborne et al. (2021). Little in this file has changed to accommodate higher dimensional neuron models. In the definition of the *Algorithm*, COND3D, the attributes *start_v, start_w*, and *start_u* allow the user to define the starting position (of a Dirac delta peak) for the population in the three-dimensional space. Similarly-named attributes can be added for higher dimensions. The *modelfile* and *transformfile* attributes should point to the required pre-processed files generated from the algorithm described in Section 2.2.

The *Connection* elements describe the inhibitory and excitatory connections between the two populations (nodes) E and I. As discussed earlier, each population simulated using the numerical PDT is influenced by one or more Poisson noise processes which change the probability mass function to approximate each neuron in the population receiving Poisson distributed spike trains. In MIIND, populations interact via their average output firing rate which becomes the rate parameter of the input Poisson process for the target population. Four such connections are set up here. The *num_connections* attribute indicates how many incoming connections each neuron in the target (*Out*) population receives from the source (*In*) population. This has the effect of multiplying the incoming firing rate parameter. The *efficacy* attribute gives the instantaneous jump value caused by a single incoming spike. The *dimension* attribute has been newly added and gives the direction in which the jump occurs. In this example, spikes from the excitatory population cause a change of 1 nS/cm^2^ change in dimension 1 which corresponds to the *w* variable. Finally, the *delay* attribute gives the transmission delay of the instantaneous firing rate between populations which allows MIIND to simulate the complex dynamics which can arise when this is a non-zero value.

All other aspects of the file remain unchanged though the *Display* element which tells MIIND to render the probability mass function of population E during the simulation now causes a three-dimensional rendering of the function in state space. For higher dimensions, which three dimensions to display can be chosen during simulation.

The main change to MIIND to support ND neuron models is the addition of the *generateNdGrid* method in the MIIND Python module. Listing 3 shows a function set up in Python, *cond*, which describes the time evolution of a LIF neuron with excitatory and inhibitory conductances. The *generateNdGrid* method generates the *cond3d.model* and *cond3d.tmat* support files which are referenced in the simulation file above (listing 2). The method takes as parameters:

1. The Python function defining the model dynamics.
2. The name of the generated files.
3. The minimum values in state space.
4. The span of the grid in state space.
5. The resolution of the grid.
6. The threshold potential.
7. The reset potential.
8. Any additional change in state of a neuron after being reset to the reset potential (in this case, there is none).
9. The timescale of the neuron model in seconds.
10. The time step with which to solve the neuron model in seconds.

**Listing 3 An example Python script to generate the support files for a three-dimensional LIF neuron population in MIIND**.


  **import** miind.miindgen as miindgen
  
  **def** cond(y):
      V_l = -70.6
      V_e = 0.0
      V_i = -75
      C = 281
      g_l = 0.03
      tau_e = 2.728
      tau_i = 10.49
  
      v = y[2]
      w = y[1]
      u = y[0]
  
      v_prime = (-g_l*(v - V_l) - w * (v - V_e) -
          u * (v - V_i)) / C
      w_prime = -(w) / tau_e
      u_prime = -(u) / tau_i
  
      **return** [u_prime, w_prime, v_prime]
  
  miindgen.generateNdGrid(cond, ’cond3d’,
      [-0.2,-0.2,-80], [5.4,5.4,40.0],
      [50,50,50], -50.4, -70.6, [0.0,0.0,0.0], 1,
      0.001)


Running a script such as this performs the steps outlined in Section 2.2. To see further examples of ND simulations in MIIND, once the software has been installed (using pip install miind), the *examples/model_archive* directory of the MIIND repository contains the required files for a number of different three- and four-dimensional neuron model populations. The three experiments presented below are available in the *examples/miind_nd_examples* directory of the MIIND repository.

### 2.4. Testing a single population

**Figure 6:**
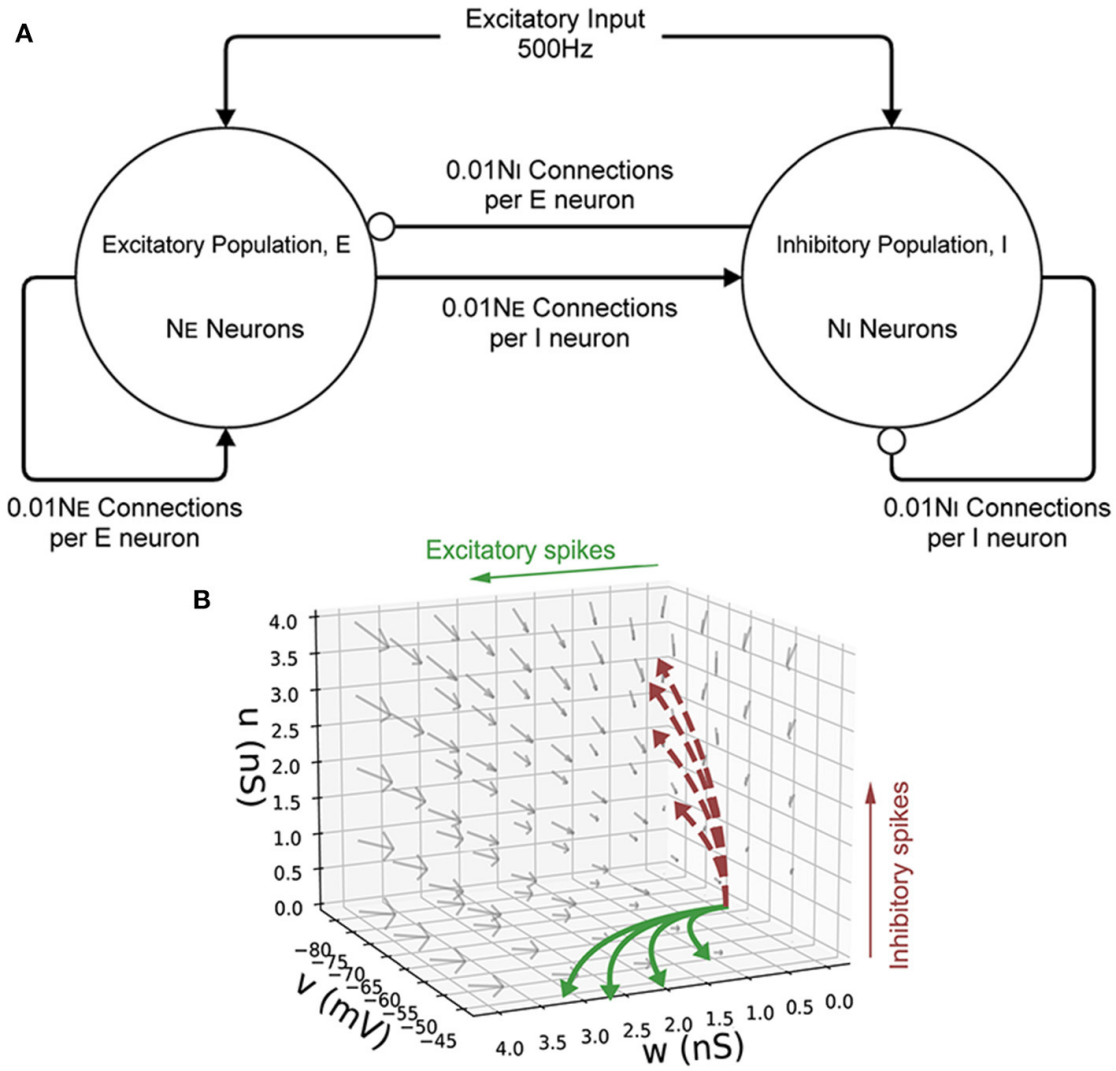
**(A)** A schematic of the E-I population network. The excitatory population, E is made up of *N*_*E*_ neurons. The inhibitory population, I contains *N*_*I*_ = 10, 000−*N*_*E*_ neurons. Each population receives an excitatory external input of 500 Hz. Each neuron in both populations receives 0.01*N*_*E*_ excitatory connections and 0.01*N*_*I*_ inhibitory connections. Arrows represent an excitatory connection, circles represent an inhibitory connection. **(B)** The three-dimensional state space of the leaky integrate-and-fire neuron with an excitatory and inhibitory synaptic conductance. *v* is the membrane potential, *w* is the excitatory synaptic conductance, and *u* is the inhibitory synaptic conductance. The vector field shows the direction of motion in state space for neurons with no external impulse. Neurons which receive an excitatory input spike are shifted higher in *w*. Neurons which receive an inhibitory input spike are shifted higher in *u*. The solid curves show trajectories of neurons under excitatory impulse alone. The dashed curves show trajectories of neurons under inhibitory impulse alone.

Initially, a single population of leaky integrate-and-fire neurons with excitatory and inhibitory synaptic conductance variables was simulated in MIIND and compared to a so-called Monte Carlo approach. The definition of the underlying neuron model is given in Equation (1) and the parameters are listed in Table 2. *v* represents the membrane potential, *u* represents the conductance of inhibitory synapses which will increase with increased inhibitory input. *w* represents the conductance of the excitatory synapses. *C* is the membrane capacitance and *g*_*l*_ is the leak conductance. *V*_*l*_, *V*_*e*_, and *V*_*i*_ are the reversal potentials for their respective conductances. The refractory period, during which the state is held constant at the reset potential, has been set to 2 ms. Figure 6 shows a schematic of the neuron model state space in three dimensions and the effect of excitatory and inhibitory input spikes. Due to the dynamics of the model, mass in cells with a high *u* value will move to lower values of *v* and mass at high *w* values will move to higher cells in *v*.


$$\begin{array}{r} C\frac{dv}{dt}=-g_{l}\left(v-V_{l}\right)-w\left(v-V_{e}\right)-u\left(v-V_{i}\right)\\ \tau_{e}\frac{w}{dt}=-w\\ \tau_{i}\frac{u}{dt}=-u \end{array}$$

(1) $$\begin{array}{r} v>threshold\to v=reset \end{array}$$

The Monte Carlo simulation was set up in Python for a population of 10,000 neurons following the dynamical system in Equation (1). For a time step of 1 ms, neurons receive a number of input spikes sampled from a Poisson distribution with a given rate parameter. Each spike causes a 1.5 nS/cm^2^ increase in the excitatory synaptic conductance variable, *w*. Each neuron also receives excitatory and inhibitory Poisson noise at 50 Hz, again, with each excitatory spike causing a 1.5 nS/cm^2^ increase in *w* and each inhibitory spike causing a 1.5 nS/cm^2^ increase in *u*. Both *u* and *w* were set to 0 nS/cm^2^ at the start of the simulation.

**Table 2:** Parameters used for Equations (1) and (2).

**Parameter name**	**Values and notes**
**Equation (1)**	**Leaky integrate-and-fire neuron with an excitatory and inhibitory synaptic conductance**
*g*_*l*_	0.03 nS/cm^2^
*E*_*l*_	−70.6 mV
*E*_*e*_	0.0 mV
*E*_*i*_	−75 mV
*C*	281 pF/cm^2^
τ_*e*_	2.728 ms
τ_*i*_	10.49 ms
Refractive period	2 ms
Threshold potential	−50.4 mV
Reset potential	−70.6 mV
**Equation (2)**	**Hodgkin-Huxley Neuron**
*g*_*l*_	0.5 mS/cm^2^
*g*_*k*_	30 mS/cm^2^
*g*_*na*_	100 mS/cm^2^
*V*_*k*_	−90 mV
*V*_*na*_	50 mV
*V*_*l*_	−65 mV
*C*	1.0 μF/cm^2^
α_*m*_	$0.32\left(13-v+V_{t}\right)/\left(e^{\frac{13-v+V_{t}}{4}}-1\right)$
α_*n*_	$0.032\left(15-v+V_{t}\right)/\left(e^{\frac{15-v+V_{t}}{5}}-1\right)$
α_*h*_	$0.128e^{\frac{17-v+V_{t}}{18}}$
β_*m*_	$0.28\left(v-V_{t}-40\right)/\left(e^{\frac{v-V_{t}-40}{5}}-1\right)$
β_*n*_	$0.5e^{\frac{10-v+V_{t}}{40}}$
β_*h*_	$4/\left(1+e^{\frac{40-v+V_{t}}{5}}\right)$
*V*_*t*_	-63 mV

A MIIND simulation was similarly set up. Six separate grid transition files were generated all according to Equation (1) but with different grid resolutions: 50 × 50 × 50 (for *u*, *w*, and *v*, respectively), 100 × 100 × 100, 150 × 150 × 150, 100 × 100 × 200, 200 × 200 × 100, and 50 × 50 × 300. For all resolutions, the grid spans the model state space for *u* = −0.2 nS/cm^2^ to 5.2 nS/cm^2^, *w* = −0.2 nS/cm^2^ to 5.2 nS/cm^2^, and *v* = −80 to −40 mV. These ranges represent the limits of the values that the variables can take in the MIIND simulation but were chosen because all significant probability mass is contained in this volume throughout. All simulations produced 1.2 s of activity. The average membrane potential, synaptic conductances, and firing rate of the population were recorded.

Though MIIND has not been fully benchmarked, it is instructive to see the relative benefits to computational efficiency with differing grid resolutions. For the grid resolutions, 50 × 50 × 50, 100 × 100 × 100, 150 × 150 × 150, and 50 × 50 × 300, the time from starting the MIIND program to the beginning of the simulation was recorded to give an indication of the effect of load times with greater transition file sizes. Then the time to complete the simulation was recorded. The same simulation from above was performed without recording the membrane potential or firing rate to the hard drive. The machine used to produce the results has a solid state drive (SSD), an Intel(R) Core(TM) i7-8750H CPU @ 2.20GHz, and an NVidia Geforce GTX 1060.

### 2.5. An E-I network

To demonstrate how MIIND is able to simulate the interaction of multiple populations and capture changes in behavior with different parameters, a population network was set up in an E-I configuration (Brunel, 2000). Figure 6 shows the population level connections. In both the MIIND and Monte Carlo simulations, for each connection, the average firing rate of the source population is used as the rate parameter for the Poisson input to the target population. The Monte Carlo simulation was set up in Python for 10,000 neurons following the dynamics of Equation (1). Parameters for the neuron model and E-I network model are adapted from Sukenik et al. (2021). The 10,000 neurons are shared among the two populations according to a ratio parameter of excitatory to inhibitory neurons. That is, the number of inhibitory neurons, *N*_*I*_ was chosen and the number of excitatory neurons, *N*_*E*_ was set equal to 10, 000−*N*_*I*_. The excitatory and inhibitory conductance jump values are held constant and a weight is multiplied by the Poisson rate parameter of each connection to reflect that each neuron should receive 0.01*N*_*E*_ excitatory connections and 0.01*N*_*I*_ inhibitory connections. A transmission delay of 3 ms is applied to all inter-population connections. Finally, each population receives a 500 Hz excitatory Poisson distributed input with each spike causing a 1.5 nS/cm^2^ jump in *w*. Table 3 gives the full list of parameters for the E-I model. MIIND was set up in the same way using a newly generated grid with resolution 150 × 150 × 150. The grid for this simulation covers a much larger volume of state space as it is expected that there will be large fluctuations in the conductance variables. Therefore, the size of the grid was set to *u* = −10 nS/cm^2^ to 100 nS/cm^2^, *w* = −5 nS/cm^2^ to 25 nS/cm^2^, and *v* = −80 to −40 mV. Across simulation trials, all parameters were kept constant except for *N*_*I*_.

**Table 3:** Parameters used for the E-I network model.

**Parameter name**	**Values and notes**
	Parameters apply to both the MIIND and Monte Carlo simulations
External firing rate	500 Hz to both E and I populations
External excitatory jump	1.5 nS/cm^2^ change in *w* per incoming spike
*N*_*I*_	Free parameter in the range 1,000–9,000
*N*_*E*_	10, 000−*N*_*I*_
Number of E to E connections	0.01*N*_*E*_
Number of E to I connections	0.01*N*_*E*_
Number of I to I connections	0.01*N*_*I*_
Number of I to E connections	0.01*N*_*I*_
Excitatory jump for E to E connections	1 nS/cm^2^ increase in *w* per incoming spike
Excitatory jump for E to I connections	1 nS/cm^2^ increase in *w* per incoming spike
Inhibitory jump for I to I connections	4 nS/cm^2^ increase in *u* per incoming spike
Inhibitory jump for I to E connections	4 nS/cm^2^ increase in *u* per incoming spike
E to E transmission delay	3 ms
E to I transmission delay	3 ms
I to I transmission delay	3 ms
I to E transmission delay	3 ms

### 2.6. A four-dimensional neuron population

To test the performance of MIIND with populations of four-dimensional neurons, we simulated a population of Hodgkin-Huxley neurons (Hodgkin and Huxley, 1952). This gold-standard model has not been simulated with a population density approach before. A fourth time-dependent variable significantly increases the amount of computation required to generate the transition matrix and its size beyond the three-dimensional case above. As before, a Monte-Carlo simulation was set up for comparison. The Hodgkin-Huxley neuron model is defined in Equation (2). As in Equation (1), the neuron has a capacitance, *C*, and a leak conductance, *g*_*l*_, with reversal potential, *V*_*l*_. The potassium and sodium synaptic conductances, *g*_*k*_ and *g*_*na*_ remain constant with respective reversal potentials, *V*_*k*_ and *V*_*na*_. However, they are modulated by the three time dependent gating variables, *n*, *m*, and *h*. The definitions of α and β are given in Table 2.


(2) $$\begin{array}{r} C\frac{dv}{dt}=-g_{k}n^{4}\left(v-V_{k}\right)-g_{na}m^{3}h\left(v-V_{na}\right)-g_{l}\left(v-V_{l}\right)\\ \frac{m}{dt}=\alpha_{m}\left(1-m\right)-\beta_{m}m\\ \frac{n}{dt}=\alpha_{n}\left(1-n\right)-\beta_{n}n\\ \frac{h}{dt}=\alpha_{h}\left(1-h\right)-\beta_{h}h. \end{array}$$

The population was given a Poisson distributed input at various rates between 0 and 40 Hz. The number of input connections to each neuron in the population was set at 100 and can be considered a weight so that the incoming rate would be multiplied by this amount. Each incoming spike produces a 3 mV jump in membrane potential. For MIIND, only one Hodgkin-Huxley grid was generated with dimensions 50 × 50 × 50 × 50 for *h*, *n*, *m*, and *v*, respectively. This resolution was chosen to keep the total number of cells low. The size of the grid was set between −0.1 and 1.1 for the gating variables, and *v* = −100 to 60 mV.

## 3. Results

### 3.1. A single population of three-dimensional neurons

**Figure 7:**
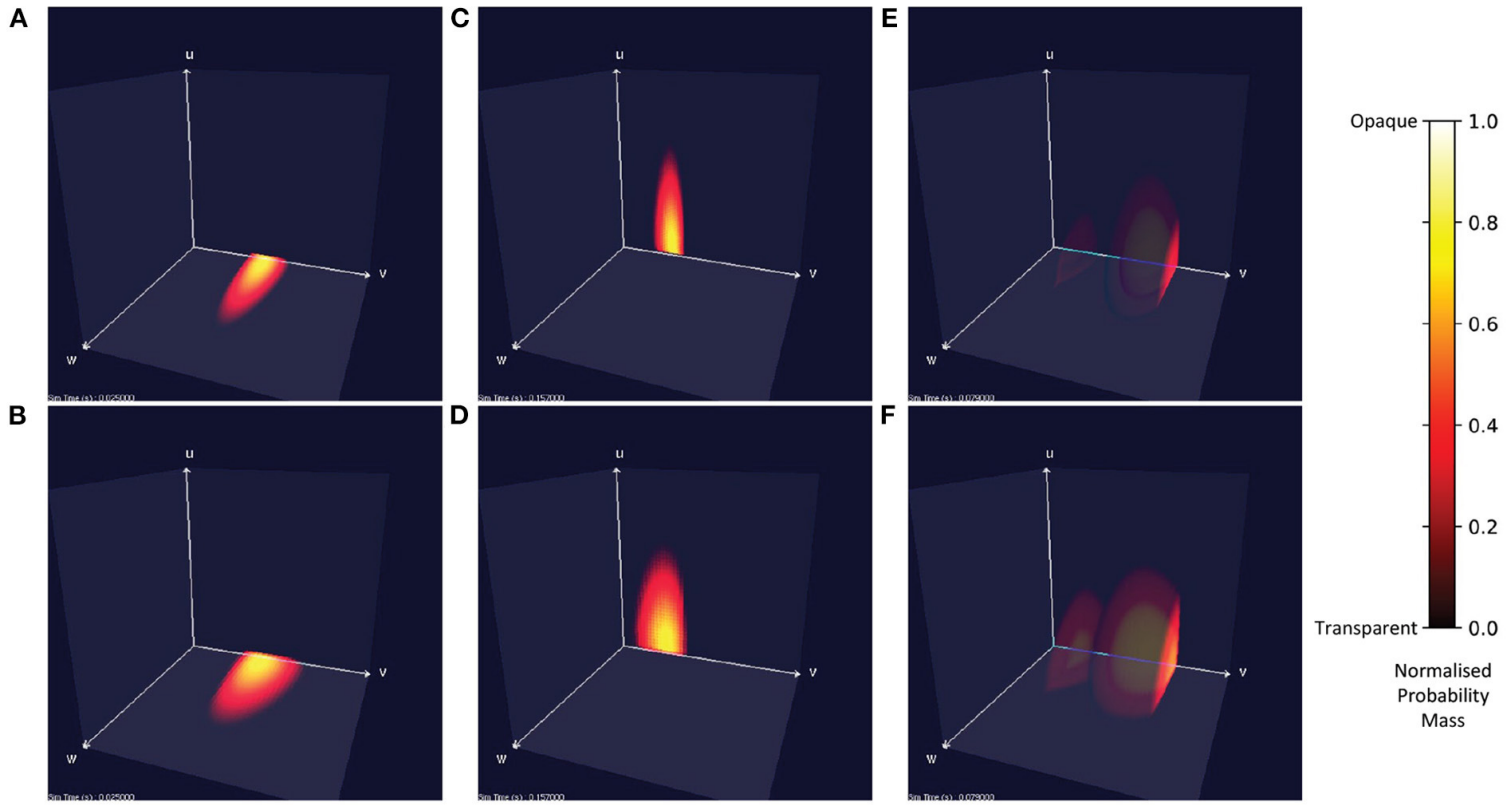
Visualizations of a population of leaky integrate-and-fire neurons with an excitatory and inhibitory synaptic conductance in MIIND. Cells with no probability mass are transparent. With increasing probability mass, they become more opaque and change from red to yellow. The color and opacity are normalized to the value of the cell with the highest probability mass. **(A,C,E)** The probability mass function across a 150 × 150 × 150 grid. **(B,D,F)** The probability mass function across a 50 × 50 × 50 grid for the same simulation time as the image above. **(A,B)** When the population receives only excitatory incoming spikes, the probability mass function remains in the plane at *u* = 0. **(C,D)** When the population receives only inhibitory incoming spikes, the probability mass function stays in the plane at *w* = 0. **(E,F)** When the population receives both inhibitory and excitatory incoming spikes, the probability mass function extends into the state space. In this case, the excitatory input is enough to overcome the inhibitory input and the mass function moves across the threshold potential. The bright face shows the probability mass at the threshold. Probability mass which has been reset reappears at the reset potential and moves further into the state space.

Figure 7 shows the probability mass functions for six different simulations of a population of leaky integrate-and-fire neurons with excitatory and inhibitory synaptic conductances. Each cell has a color/brightness and an alpha or transparency value such that cells with a higher probability mass are a brighter yellow, and more opaque than cells with lower probability mass which are darker red and more transparent. This plotting style allows the center of the function volume to be seen from the outside. Cells with zero probability are entirely transparent so that only significant cells are visible. Due to the greater opacity which often appears in the central volume of the function, the MIIND user may also rotate the entire volume to view the function from all angles. In Figures 7A,B, when only an excitatory input is provided, the function remains in the two-dimensional plane at *u* = 0 and is the same function as produced in the purely two-dimensional model demonstrated in de Kamps et al. (2019) and Osborne et al. (2021). Likewise, when only an inhibitory input is provided (Figures 7C,D), the function stays at *w* = 0. Figures 7E,F show the result of both an excitatory and inhibitory input. When enough excitatory input is provided, probability mass reaches the threshold membrane potential and is reset causing a sharp cut-off at those values. The brighter yellow cells in the center of the function's volume indicate that the majority of neurons can be found there traveling from the reset to threshold potential receiving close to the average number of excitatory and inhibitory input spikes. Further out, at higher values of *u* and *w*, the probability of finding a neuron reduces as neurons are less likely to receive many more spikes than average.

**Figure 8:**
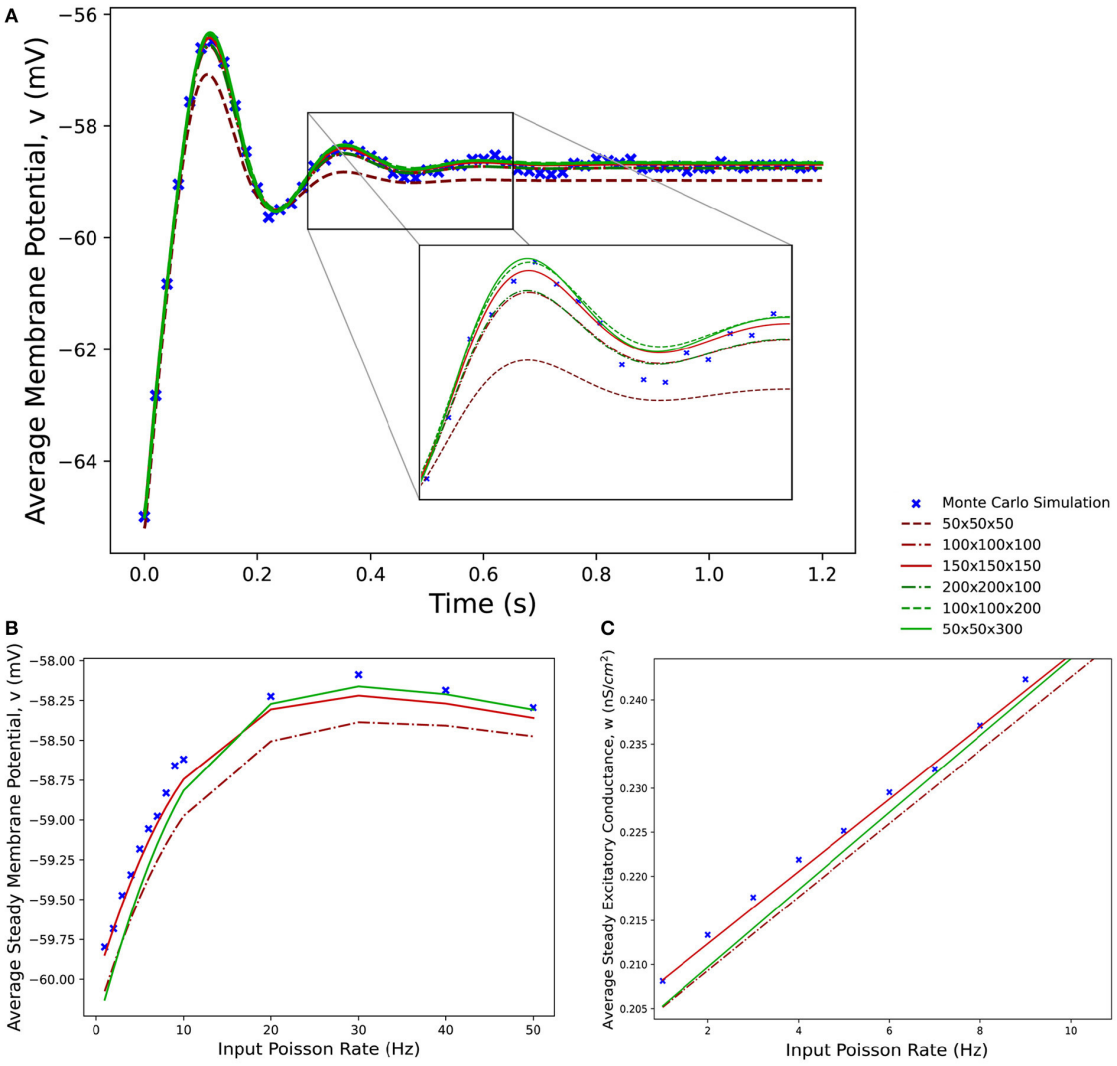
**(A)** The average membrane potential for a single population of leaky integrate-and-fire neurons with excitatory and inhibitory synaptic conductances simulated using a Monte Carlo approach and using MIIND with grids of different resolutions. **(B)** The effect on the average steady state membrane potential with different rates of the Poisson distributed input for the Monte Carlo simulation and different MIIND grid resolutions. **(C)** The effect on the average steady state excitatory conductance variable with increasing Poisson input rate. Only the mean of the values for the Monte Carlo simulation are shown here (without a variance or standard deviation) because the MIIND simulation produces no such statistic and so no comparison can be made.

Figure 8 shows average membrane potential recorded from multiple simulations of a population of leaky integrate-and-fire neurons with excitatory and inhibitory synaptic conductances. The scatter points show the average potential of 10,000 individual neurons simulated using the Monte Carlo approach. The remaining curves show the average potential of populations simulated in MIIND using 3-dimensional grids of different resolutions. For the transient period before the membrane potential reaches a steady state, all the MIIND simulations remain synchronized with the Monte Carlo results. As would be expected, the least accurate result comes from the lowest resolution grid, 50 × 50 × 50. However, even at this resolution, the mean error between the Monte Carlo activity and the MIIND result is only 0.354 mV. The error is reduced significantly for 100 × 100 × 100 (0.115 mV) then further reduced but only slightly for 150 × 150 × 150 (0.063 mV) suggesting a degree of diminishing return for increasing the resolution in an equal fashion across dimensions. The error from the 200 × 200 × 100 grid is the same as the 100 × 100 × 100 grid but the 100 × 100 × 200 grid does better (0.059 mV) indicating that increasing the resolution of the membrane potential dimension is a more efficient way to attain accurate results for this underlying neuron model. To illustrate this further, the 50 × 50 × 300 grid performs the best of the trials with an average error of 0.054 mV despite the low resolution of the conductance dimensions. Over a range of average rates (Figure 8B) of the Poisson distributed input, the steady state membrane potential of the MIIND simulations, again, approaches those of the Monte Carlo results with increasing resolution. For low input rates, when the majority of neurons are subthreshold, the 150 × 150 × 150 grid gives the closest approximation to the Monte Carlo results. However, once the majority of neurons are crossing the threshold and firing, the 50 × 50 × 300 grid gives better agreement. Figure 8C shows the average excitatory conductance variable for the grids across the range of lower input rates (1–10 Hz) in comparison to the Monte Carlo approach. The 50 × 50 × 300 grid underestimates the conductance which could account for the underestimation of the membrane potential for the same input. The 150 × 150 × 150 grid, by contrast, has better agreement with the membrane potential and excitatory conductance for these rates which produce mostly sub-threshold activity in the population.

**Figure 9:**
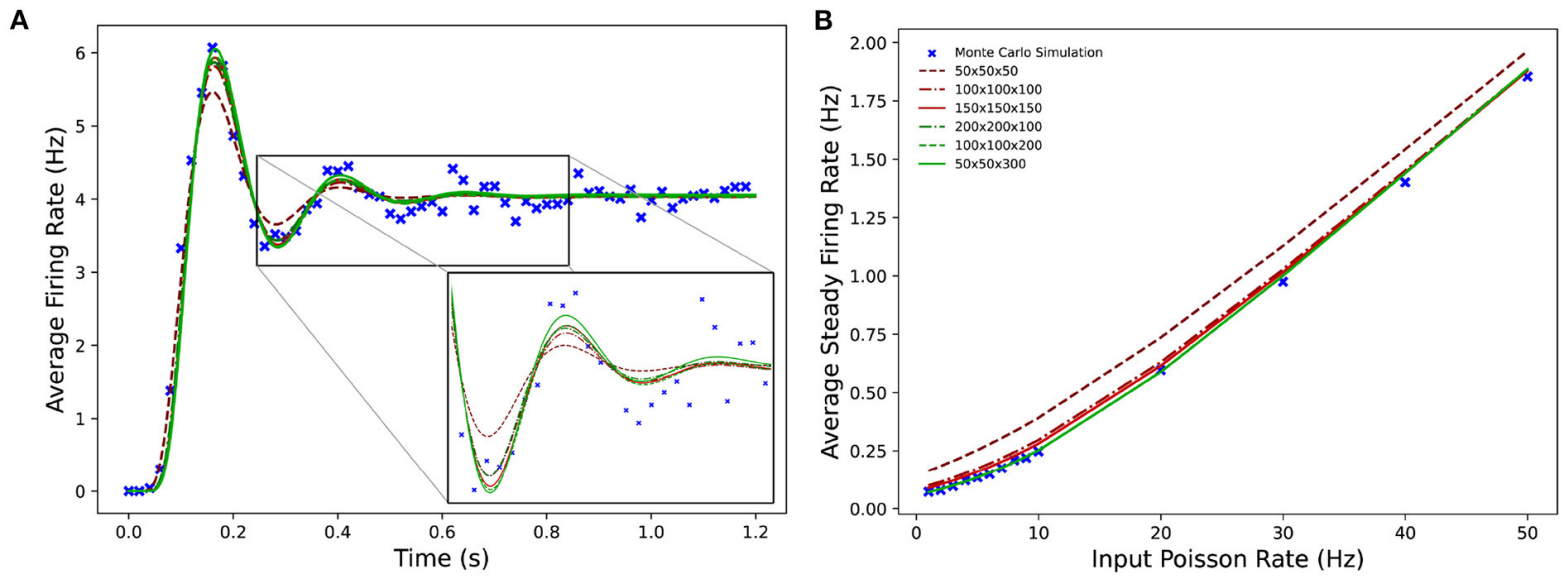
**(A)** The average firing rate of a single population of leaky integrate-and-fire neurons with excitatory and inhibitory synaptic conductances simulated using a Monte Carlo approach and using MIIND with grids of different resolutions. **(B)** The effect on the average steady state firing rate of the population with increasing rate of the Poisson distributed input.

Figure 9 shows the average firing rates of the same Monte Carlo and MIIND simulations. The differences in grid resolution produce a similar trend in error, with the lowest resolution, 50 × 50 × 50 laying furthest away from the Monte Carlo simulation and the 50 × 50 × 300 grid the closest. However even at lower resolutions, all the average firing rates of the MIIND populations are very well matched to direct simulation.

### 3.2. Simulation speed for different grid resolutions

Table 4 shows the load and simulation times for 1 s of a single population of leaky integrate-and-fire neurons with excitatory and inhibitory synaptic conductances. As expected, as the total number of cells increases the load times and simulation times increase. When running multiple short simulations, the load time becomes a significant consideration. However, only the simulation time is dependent on the required length of the simulation. The load time remains constant.

**Table 4:** Times to simulate 1 s of a population of leaky integrate-and-fire neurons with excitatory and inhibitory synaptic conductances in MIIND using different grid resolutions.

**Grid resolution**	**Time to load the grid (s)**	**Time to run the simulation (s)**
50 × 50 × 50	4.82	2.71
100 × 100 × 100	35.58	15.18
150 × 150 × 150	126.01	48.7
50 × 50 × 300	27.62	11.93

### 3.3. Three-dimensional neurons in an E-I population network

**Figure 10:**
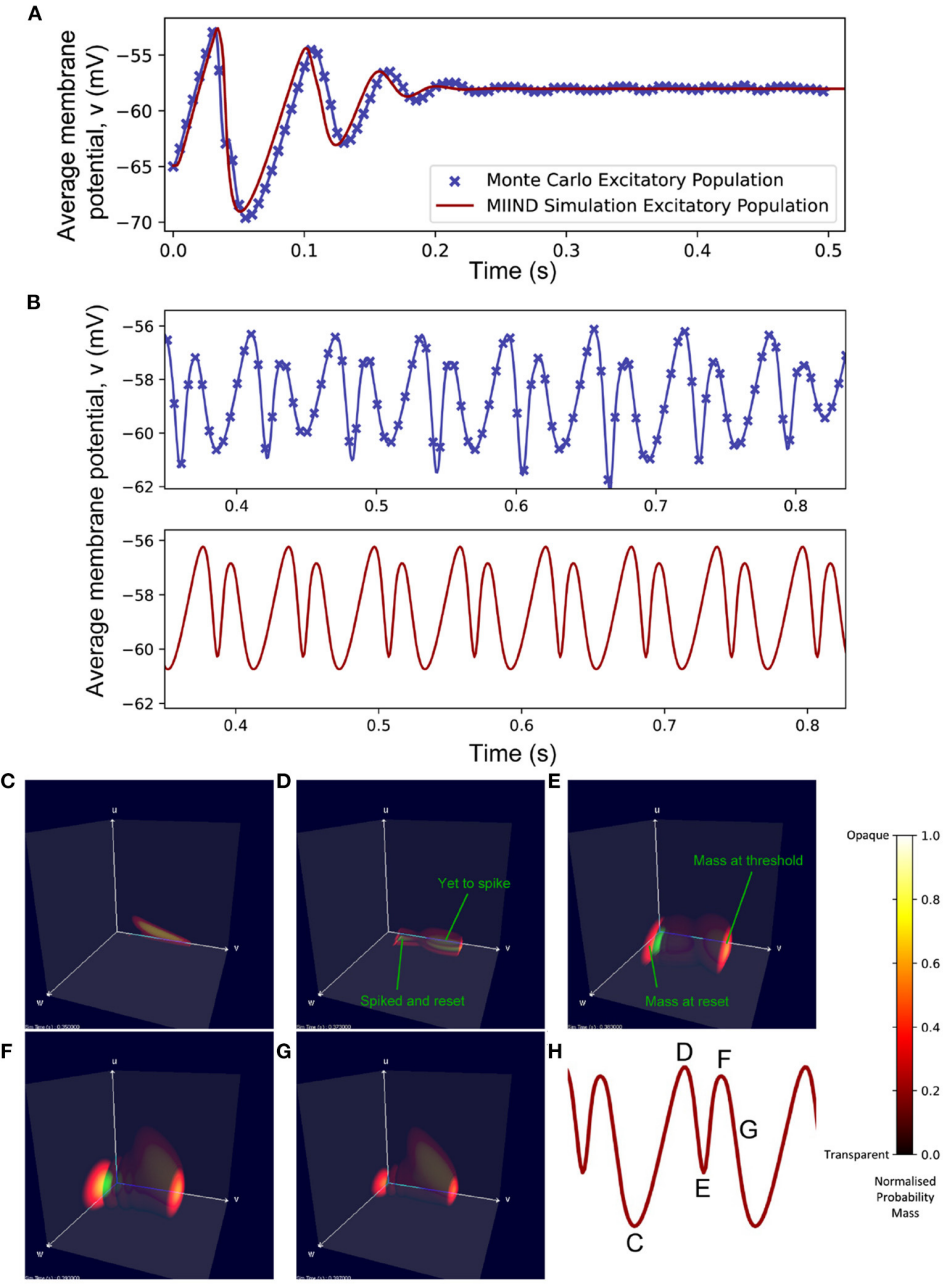
**(A)** The average membrane potential of the excitatory population in the E-I network with a ratio of 1:1 excitatory and inhibitory neurons (*N*_*E*_ = *N*_*I*_). In the MIIND simulation, each connection between populations has the “number of connections” value set to 50. **(B)** The average membrane potential of the excitatory population in the E-I network with a ratio of 8:2 excitatory to inhibitory neurons (*N*_*E*_ = 8, 000, *N*_*I*_ = 2, 000). In the MIIND simulation, the excitatory connections have the “number of connections” value set to 80 and the inhibitory connections have the “number of connections” value set to 20. For clarity, the traces from the Monte Carlo simulation and the MIIND simulation have been separated. **(C–H)** The probability mass function for the excitatory population in MIIND during the double peaked oscillation with a connection ratio of 8:2. **(H)** shows the corresponding points in the oscillation. At **(C)**, The population only experiences the external input of 500 Hz and is pushed toward the threshold. At **(D)**, though some probability mass has passed threshold and been reset, the majority is close to the threshold and so the average membrane potential is at a peak. At **(E)**, probability mass has continued to cross the threshold so that now a large amount is near the reset potential which brings the average back down. The function has also shifted higher in *w* due to the excitatory self-connections and more probability mass is pushed across threshold. The function also begins moving upwards in *u* from the increased inhibitory input but this is not enough to overcome the excitation. At **(F)**, the inhibitory input has continued to push the probability mass function higher in *u* and much less probability mass now crosses the threshold. At **(G)**, the function continues to shift back away from threshold approaching **(C)** once again.

For the Monte Carlo simulation of 5,000 excitatory and 5,000 inhibitory neurons (with an average of 50 excitatory and 50 inhibitory incoming connections to each), the two populations reach an equilibrium state after an initial transitory phase. Figure 10A shows excellent agreement between the average membrane potentials from the two approaches. The initial oscillation in the transient period covers nearly 100 nS/cm^2^ in *u* and 12 nS/cm^2^ in *w* which requires a much larger volume of state space than the single population simulation because of the large synaptic efficacies and recurrent connections involved in the E-I network. In MIIND, as the oscillations reduce, the state space covered by the probability mass function reduces and is therefore discretized by fewer cells. In other words, the cell density covering the function is lower. However, this only causes a minimal amount of additional damping to the oscillation as the function reaches equilibrium.

In the Monte Carlo simulation, with 8,000 excitatory neurons and 2,000 inhibitory neurons, both populations produce an oscillating pattern as shown in Figure 10B. In the MIIND simulation, in order to match the connection ratios between populations, the number of excitatory and inhibitory connections is set to 80 and 20, respectively. The simulation is also able to produce a similar oscillatory pattern. As would be expected, the population density approach produces a regular oscillation while the Monte Carlo has some variation in the length and amplitude of each oscillation. The double peak of each oscillation can be explained by observing the probability mass function in MIIND during the simulation (Figures 10C–H). The initial peak is produced as the whole population depolarizes and approaches the threshold potential. As mass begins to pass the threshold, the reset mass brings the average membrane potential back down. The probability mass is pushed higher in *w* and *u* as the recurrent excitatory input and inhibitory input from the other population increase. The excitatory input has the strongest effect on the probability mass close to the reset potential which begins to push the average membrane potential back up toward a second peak. The inhibitory input has the strongest effect on the probability mass close to threshold and less and less mass reaches threshold. The split probability mass function coalesces once more and the cycle can repeat.

When the ratio of excitatory to inhibitory neurons is 9:1, the Monte Carlo simulation demonstrates how the excitatory self-connection causes the excitatory population activity to “blow-up” such that the excitatory conductance reaches a maximum value and neurons fire at their maximum rate. This state is a challenge for MIIND to emulate. Firstly, the number of iterations required to solve the Poisson master equation each time step must be increased to 1,000 due to the instability caused by such high firing rates. Secondly, the excitatory conductance variable frequently approaches 80 nS/cm^2^ and so the grid must cover a large amount of state space requiring an unreasonable resolution in the *w* dimension to maintain the same cell density as previous simulations.

### 3.4. A single population of four-dimensional Hodgkin Huxley neurons

**Figure 11:**
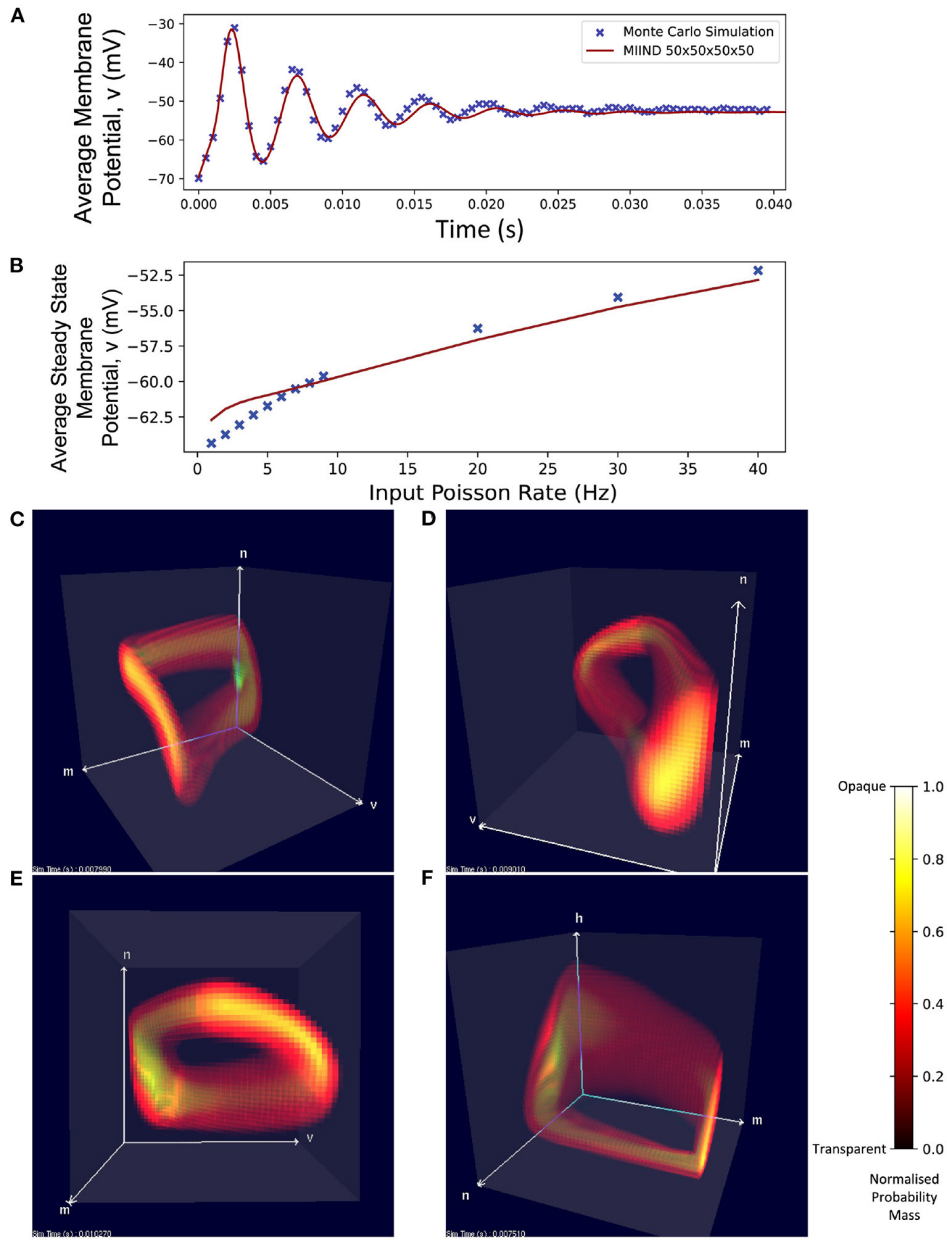
**(A)** The average membrane potential of a single population of Hodgkin-Huxley neurons simulated using a Monte Carlo approach and in MIIND with a 50 × 50 × 50 × 50 grid. **(B)** The average steady state membrane potential with different rates of the Poisson distributed input. **(C–E)** The three-dimensional marginal probability mass function of the four-dimensional Hodgkin-Huxley neuron population in MIIND having reached a steady state. The membrane potential *v*, sodium activation variable *m*, and potassium activation variable *n* are shown. **(F)** The three-dimensional marginal probability mass function showing the gating variables, *w*, *n*, and *h* (sodium inactivation variable) only.

Even with a low resolution of 50 × 50 × 50 × 50, MIIND is able to simulate the probability mass function of a population of Hodgkin Huxley neurons and achieve good agreement with the transient activity and steady state membrane potential of an equivalent Monte Carlo simulation (Figure 11A). Figure 11B shows how, for a range of input firing rates, the resulting average membrane potential at steady state approximates that of the Monte Carlo simulations better for higher frequencies. At low input rates, the membrane potential is overestimated. MIIND displays the probability mass function for three of the four dimensions at a time as shown in Figure 11C. With a key press, the user can change the order of dimensions displayed and see any combination of variables. Figure 11F shows the three gating variables, *m*, *n*, and *h*.

## 4. Discussion

The original motivation for applying a population density approach to simulate neurons was to reduce the computational complexity when analyzing a large population of homogeneous neurons. This has since been made somewhat redundant with the development of more powerful computers and especially the use of GPGPU architectures. For example, GeNN (Yavuz et al., 2016; Knight et al., 2021) can simulate in real time the well known Potjans-Diesmann microcircuit model (Potjans and Diesmann, 2014) which comprises around 10,000 neurons. The analytical solution for the behavior of a leaky integrate-and-fire population developed by Omurtag et al. (2000) using the diffusion approximation would undoubtedly prove efficient, requiring only a single calculation per population per time interval. However, it would require a lot of manual work to define the full population network and if a different neuron model were to substitute the integrate-and-fire neuron, the entire solution would need to be re-derived. Even with newer techniques such as the refractory density approach, work is required to get the underlying neuron model in a form that can be processed. MIIND uniquely overcomes this limitation allowing the user to define the neuron model without any further manual process to produce a numerical solution to the population density approach. However, solving the master equation for the non-deterministic noise component of the dynamics requires repeated applications of the jump transitions shown in Figure 3. As discussed by Osborne et al. (2021), depending on the model, the time step, and the input firing rate, solving the master equation can require tens or hundreds of iterations per time step of the simulation. This was the case for the EI network in the 8:2 ratio. The sharp changes in firing rate of the two populations combined with large synaptic conductance jumps meant that solving the master equation required 100 iterations per cell per time step to remain stable. The numerical population density approach in MIIND should, therefore, not be used for simulations where computational speed is the most important factor. However, it has been shown (de Kamps et al., 2019) that there is at least an order of magnitude improvement in memory consumption over direct simulation techniques, such as that of NEST, as there is no requirement to store the spike history.

Although computational efficiency is not the primary reason for using the population density approach, there are some benefits to generating the probability mass function over a direct simulation of individual neurons. The probability mass function can be considered the idealized distribution of neuron states. Cells in the grid which have zero mass correspond to volumes of state space where neuron states should never appear. This can be difficult to approximate with a direct simulation of individual neurons for parts of the distribution with a low but non-zero probability mass. Inconsistencies between the behavior of real neurons and a model could be identified more effectively by comparing to the probability mass function. Also due to the idealized probability mass function, the output metrics of a population such as average firing rate and average membrane potential have no variation due to noise or a specific realization of the Poisson distributed input. Therefore, no averaging or smoothing is required to produce more readable results as would be expected from a direct simulation (Figures 8, 9). In the E-I network, 10,000 Monte Carlo neurons was enough to produce a similar result to the MIIND simulation. But when that number is reduced to 1,000, there is greater variation in the firing rate and average membrane potential of the population. In the E-I network, a temporarily high number of spikes from the excitatory population leads to increased excitatory input 3 ms later and increased inhibitory input 3 ms after that. The resulting reduction in average membrane potential and firing rate is therefore exaggerated which produces an overall skew of these metrics. A population in MIIND can be thought of as an infinite number of trials of a single neuron or as an infinite number of neurons performing a single trial once. Because of this, a MIIND simulation is independent of the number of neurons in the population and cannot produce so-called finite size effects. This can be a useful feature as it is not always as clear from a Monte Carlo simulation what behavior stems from the finite size and what is a population level effect.

Finally, the visualization of the probability mass function in MIIND could prove to be a valuable educational tool for understanding the behavior of neural populations under the influence of random spikes. In fact, any N-dimensional dynamical system under the influence of shot noise could be observed although this has not been attempted. It would be easy enough to plot points in a three-dimensional state space for individually simulated neurons but points at the front of the distribution would obscure those at the back and in the center. Producing a smooth enough distribution and to pick an appropriate transparency value for each cell would require a population of millions of neurons.

The increased time to produce the probability mass function over direct simulation does not negate the usefulness of lower resolution grids to improve the simulation time as shown in Table 4. In particular, using a low resolution grid can greatly improve workflow when designing or prototyping a new model. Building a model which performs as required involves multiple runs of the simulation as parameters are adjusted or when errors are identified. This is another reason why it is convenient that MIIND renders each population's probability mass function while the simulation is running. As shown in Figure 11, viewing the probability mass function across all dimensions from any angle as the simulation progresses gives both insight into how the population behaves and any unexpected behavior is quickly identified.

### 4.1. What is the theoretical output spike distribution of a population in MIIND?

Different populations in MIIND interact via their average firing rates. For each connection, the average firing rate of the source population is taken as the rate parameter to a Poisson distributed input to the target population. For a one-dimensional neuron model such as a leaky integrate-and-fire neuron for which incoming spikes cause an instantaneous jump in membrane potential, it is reasonable to assume that neurons in the population are pushed over threshold directly and only due to the Poisson distributed input suggesting that the output distribution should also be Poisson distributed. However, in higher dimensional models with, for example, the addition of excitatory and inhibitory synaptic conductances, it becomes clear that neurons can move across threshold without direct influence from the Poisson input. If a sample of neurons are taken from the probability mass distribution at the beginning of a simulation, by definition, the probability that each sampled neuron is above threshold in a given time step is the probability mass which sits above threshold to be transferred to the reset potential. As the behavior of all neurons are independent by virtue of being unconnected and homogeneous, the distribution of spiking neurons from the population at each time step can be considered binomial with *p* equal to the total probability mass above threshold. Using the average firing rate as the parameter to a Poisson input for each population is therefore a reasonable approximation. Models such as the E-I network which have self-connections and loop-connections invalidates the assumption of independence and further work is required to assess if using Poisson distributed outputs is appropriate under such circumstances.

### 4.2. Finite size populations

The main function of MIIND is to use the numerical population density approach to simulate population behavior. However, a population of finite size can also be simulated which makes use of the transition matrix file and calculated jump transitions. This hybrid version of the algorithm is closer to direct simulation. A list of M grid coordinates is stored which represents the location in state space of M individual neurons. At each time step, each coordinate is updated to one of the possible transition cells defined in the transition file with probability equal to the proportion of mass in that transition. To capture the non-deterministic dynamics, a Poisson distributed random number of spikes is sampled and the calculated jump transition is applied that many times. Again, the jump transition mass proportions are used as the probability for choosing the coordinate update with each jump. The average firing rate of the population is the number of neurons above threshold (which are then translated to the reset potential) divided by M. Currently direct connections between neurons is not implemented and instead, the average firing rate is used as the Poisson rate parameter applied to all neurons in the target population. Because the Poisson master equation is not required to solve the non-deterministic dynamics, this algorithm is much faster than the population density technique and approaches the speeds of simulations in GeNN although this has not been fully benchmarked. The two main reasons for using the finite size algorithm in MIIND are to further speed up prototyping of new models and to more easily eliminate finite-size effects.

### 4.3. Other potential models for study

The ability to easily simulate populations of three- and four-dimensional neuron models opens a world of possibilities for the population density approach. The Tsodyks-Markram synapse model (Tsodyks and Markram, 1997), for example, can be combined with a leaky integrate-and-fire neuron model to define a four-dimensional system. In the original work, the model was shown to support both rate coding between neurons and more precise spike timing based on the configuration of resource management in the synapse. For a large population, simulating the rate coding configuration makes more sense but MIIND could also be used to investigate the resilience of the spike timing configuration to noise. Booth and Rinzel (1995) developed a two-compartment minimal motor neuron model. Each compartment requires two dimensions and MIIND would therefore be able to simulate a population of both compartments together. This model can reproduce the bi-stable behavior of motor neurons such that a suitable incoming excitatory burst of spikes can shift the population to an up state where it remains even in the absence of further input. This is a candidate for identifying any finite size effects and, in the presence of noise, estimating the amplitude and duration of the required excitatory and inhibitory bursts to switch states.

### 4.4. Limitations

The population density approach suffers from the so-called curse of dimensionality. With each additional time-dependent variable in the underlying neuron model, the number of cells in the grid is multiplied by the resolution of the new dimension. Not only does this produce an exponential increase in the number of cells for which the deterministic and non-deterministic dynamics must be solved, but the number of transitions per cell in the transition file also increases in most cases. The 50 × 50 × 50 × 50 transition file for the Hodgkin Huxley model runs to nearly 1.5 Gb all of which must be loaded into graphics memory. There is still work to do to improve the memory management in MIIND but it is likely that a 5-dimensional transition matrix would not fit in the memory of current graphics hardware. In addition, generating the Hodgkin-Huxley transition file takes over 100 h on the four CPU cores of a typical PC. This is a one-time preprocessing requirement that can be mitigated somewhat with high performance computing systems but, again, for higher dimensional models the time required would become unfeasible.

In many cases, the number of cells in the grid that contain a non-zero amount of probability mass at any time during the simulation is much lower than the total number of cells. For higher dimensions it would be possible to calculate the non-deterministic dynamics transitions required for a cell when probability mass is first transferred to it during the simulation. The simulation would be considerably slower at the beginning but would approach the original speeds as more cells are calculated. The memory requirements would only come from the cells involved in the probability mass function. This adaptation would still have an upper limit on the number of dimensions as the number of involved cells would still increase with greater dimensionality but it would be far from the exponential increase currently.

Another potential method for improving performance in both memory and computation speed would be to relax the requirement that all grid cells are the same size. In areas of state space where the dynamics are expected to follow a shallow curve (as opposed to the sharp turns in state space which can occur near unstable stationary points for example), larger cells could be defined. In order to preserve the benefit of equally sized cells when calculating the jump transition, the larger cells could be subdivided at simulation time and the deterministic dynamics transitions into the large cell could be linearly interpolated throughout. While not significantly affecting the computation time, the memory requirements would improve with the reduced number of transitions.

## 5. Conclusion

We have demonstrated for the first time, a numerical population density technique to simulate populations of N-dimensional neurons. Although models of higher than 5 dimensions are currently technologically out of reach, it is a significant achievement to produce the probability mass function of a population of 4-dimensional Hodgkin-Huxley neurons and to be able to visualize it in such a fashion. Implementing this technique in MIIND results in a very low barrier to entry for new users allowing them to define their desired neuron model in Python, automatically generate the required transition files and run the simulation without expert knowledge of the technique or any involved technical knowledge beyond some basic Python and XML. Although originally conceived as a technique to improve computational efficiency when simulating large populations of neurons, the population density technique cannot achieve the speeds of some other simulation methods. However, there are a number of benefits to using it, particularly in the areas of theoretical neuroscience and as a tool for analysis.

## Data availability statement

The MIIND source code and installation packages are available as a github repository at https://github.com/dekamps/miind. MIIND can be installed for use in Python using “pip install miind” on many Linux, MacOS, and Windows machines with python versions ≥3.6. Documentation is available at https://miind.readthedocs.io/. The leaky integrate-and-fire model, E-I network model, and Hodkin-Huxley model can be found in the examples/model archive directory of the MIIND repository on github.

## Author contributions

The article and code development was undertaken by HO with support and advice from MK. All authors contributed to the article and approved the submitted version.

## Funding

This project received funding from the European Union's Horizon 2020 research and innovation programme under Grant Agreement No. 785907 (Human Brain Project SGA2) (MK). HO was funded by EPSRC (EP/N509681/1). The funders had no role in study design, data collection and analysis, decision to publish, or preparation of the manuscript.

## Conflict of interest

The authors declare that the research was conducted in the absence of any commercial or financial relationships that could be construed as a potential conflict of interest.

## Publisher's note

All claims expressed in this article are solely those of the authors and do not necessarily represent those of their affiliated organizations, or those of the publisher, the editors and the reviewers. Any product that may be evaluated in this article, or claim that may be made by its manufacturer, is not guaranteed or endorsed by the publisher.

## References

[B1] Barber C. B. Dobkin D. P. Huhdanpaa H. (1996). The quickhull algorithm for convex hulls. ACM Trans. Math. Softw. 22, 469–483. 10.1145/235815.23582132355412

[B2] Bogacz R. Brown E. Moehlis J. Holmes P. Cohen J. D. (2006). The physics of optimal decision making: a formal analysis of models of performance in two-alternative forced-choice tasks. Psychol. Rev. 113, 700. 10.1037/0033-295X.113.4.700 17014301

[B3] Booth V. Rinzel J. (1995). A minimal, compartmental model for a dendritic origin of bistability of motoneuron firing patterns. J. Comput. Neurosci. 2, 299–312. 10.1007/BF00961442 8746404

[B4] Brette R. Gerstner W. (2005). Adaptive exponential integrate-and-fire model as an effective description of neuronal activity. J. Neurophysiol. 94, 3637–3642. 10.1152/jn.00686.2005 16014787

[B5] Brown K. Q. (1979). Voronoi diagrams from convex hulls. Inform. Process. Lett. 9, 223–228. 10.1016/0020-0190(79)90074-734720304

[B6] Brunel N. (2000). Dynamics of sparsely connected networks of excitatory and inhibitory spiking neurons. J. Comput. Neurosci. 8, 183–208. 10.1023/A:1008925309027 10809012

[B7] Chizhov A. Campillo F. Desroches M. Guillamon A. Rodrigues S. (2019). Conductance-based refractory density approach for a population of bursting neurons. Bull. Math. Biol. 81, 4124–4143. 10.1007/s11538-019-00643-8 31313084

[B8] Chizhov A. V. Graham L. J. (2007). Population model of hippocampal pyramidal neurons, linking a refractory density approach to conductance-based neurons. Phys. Rev. E 75, 011924. 10.1103/PhysRevE.75.011924 17358201

[B9] de Kamps M. (2006). An analytic solution of the reentrant poisson master equation and its application in the simulation of large groups of spiking neurons, in The 2006 IEEE International Joint Conference on Neural Network Proceedings (Vancouver, BC: IEEE), 102–109. 10.1109/IJCNN.2006.246666

[B10] De Kamps M. Lepperød M. Lai Y. M. (2019). Computational geometry for modeling neural populations: from visualization to simulation. PLoS Comput. Biol. 15, e1006729. 10.1371/journal.pcbi.1006729 30830903 PMC6417745

[B11] FitzHugh R. (1961). Impulses and physiological states in theoretical models of nerve membrane. Biophys. J. 1, 445. 10.1016/S0006-3495(61)86902-6 19431309 PMC1366333

[B12] Gewaltig M.-O. Diesmann M. (2007). NEST (neural simulation tool). Scholarpedia 2, 1430. 10.4249/scholarpedia.1430

[B13] Haiman M. (1991). A simple and relatively efficient triangulation of the n-cube. Discrete Comput. Geometry 6, 287–289. 10.1007/BF02574690

[B14] Hodgkin A. L. Huxley A. F. (1952). The components of membrane conductance in the giant axon of loligo. J. Physiol. 116, 473. 10.1113/jphysiol.1952.sp004718 14946714 PMC1392209

[B15] Izhikevich E. M. (2007). Dynamical Systems in Neuroscience. Cambridge, MA: MIT Press. 10.7551/mitpress/2526.001.0001

[B16] Jansen B. H. Rit V. G. (1995). Electroencephalogram and visual evoked potential generation in a mathematical model of coupled cortical columns. Biol. Cybernet. 73, 357–366. 10.1007/BF00199471 7578475

[B17] Johannesma P. I. M. (1969). Stochastic neural activity: a theoretical investigation (Ph.D. thesis). Faculteit der Wiskunde en Natuurwetenschappen, Nijmegen, Netherlands.

[B18] Knight B. W. (1972). Dynamics of encoding in a population of neurons. J. Gen. Physiol. 59, 734–766. 10.1085/jgp.59.6.734 5025748 PMC2203203

[B19] Knight J. C. Komissarov A. Nowotny T. (2021). PyGeNN: a Python library for gpu-enhanced neural networks. Front. Neuroinform. 15, 659005. 10.3389/fninf.2021.659005 33967731 PMC8100330

[B20] Ly C. Tranchina D. (2009). Spike train statistics and dynamics with synaptic input from any renewal process: a population density approach. Neural Comput. 21, 360–396. 10.1162/neco.2008.03-08-743 19431264

[B21] Mattia M. Del Giudice P. (2002). Population dynamics of interacting spiking neurons. Phys. Rev. E 66, 051917. 10.1103/PhysRevE.66.051917 12513533

[B22] Nagumo J. Arimoto S. Yoshizawa S. (1962). An active pulse transmission line simulating nerve axon. Proc. IRE 50, 2061–2070. 10.1109/JRPROC.1962.288235

[B23] Naud R. Gerstner W. (2012). Coding and decoding with adapting neurons: a population approach to the peri-stimulus time histogram. PLoS Comput. Biol. 8, e1002711. 10.1371/journal.pcbi.1002711 23055914 PMC3464223

[B24] Nykamp D. Q. Tranchina D. (2000). A population density approach that facilitates large-scale modeling of neural networks: analysis and an application to orientation tuning. J. Comput. Neurosci. 8, 19–50. 10.1023/A:1008912914816 10798498

[B25] Omurtag A. Knight B. W. Sirovich L. (2000). On the simulation of large populations of neurons. J. Comput. Neurosci. 8, 51–63. 10.1023/A:1008964915724 10798499

[B26] Osborne H. Lai Y. M. Lepperød M. E. Sichau D. Deutz L. De Kamps M. (2021). MIIND: a model-agnostic simulator of neural populations. Front. Neuroinform. 15, 614881. 10.3389/fninf.2021.614881 34295233 PMC8291130

[B27] Potjans T. C. Diesmann M. (2014). The cell-type specific cortical microcircuit: relating structure and activity in a full-scale spiking network model. Cereb. Cortex 24, 785–806. 10.1093/cercor/bhs358 23203991 PMC3920768

[B28] Rangan A. V. Cai D. (2007). Fast numerical methods for simulating large-scale integrate-and-fire neuronal networks. J. Comput. Neurosci. 22, 81–100. 10.1007/s10827-006-8526-7 16896522

[B29] Schmutz V. Gerstner W. Schwalger T. (2020). Mesoscopic population equations for spiking neural networks with synaptic short-term plasticity. J. Math. Neurosci. 10, 1–32. 10.1186/s13408-020-00082-z 32253526 PMC7136387

[B30] Schwalger T. Chizhov A. V. (2019). Mind the last spike-firing rate models for mesoscopic populations of spiking neurons. Curr. Opin. Neurobiol. 58, 155–166. 10.1016/j.conb.2019.08.003 31590003

[B31] Schwalger T. Deger M. Gerstner W. (2017). Towards a theory of cortical columns: from spiking neurons to interacting neural populations of finite size. PLoS Comput. Biol. 13, e1005507. 10.1371/journal.pcbi.1005507 28422957 PMC5415267

[B32] Sirovich L. Everson R. Kaplan E. Knight B. O'Brien E. Orbach D. (1996). Modeling the functional organization of the visual cortex. Phys. D Nonlinear Phenomena 96, 355–366. 10.1016/0167-2789(96)00033-4

[B33] Sukenik N. Vinogradov O. Weinreb E. Segal M. Levina A. Moses E. (2021). Neuronal circuits overcome imbalance in excitation and inhibition by adjusting connection numbers. Proc. Natl. Acad. Sci. U.S.A. 118:e2018459118. 10.1073/pnas.2018459118 33723048 PMC8000583

[B34] Tsodyks M. V. Markram H. (1997). The neural code between neocortical pyramidal neurons depends on neurotransmitter release probability. Proc. Natl. Acad. Sci. U.S.A. 94, 719–723. 10.1073/pnas.94.2.719 9012851 PMC19580

[B35] Wilson H. R. Cowan J. D. (1972). Excitatory and inhibitory interactions in localized populations of model neurons. Biophys. J. 12, 1–24. 10.1016/S0006-3495(72)86068-5 4332108 PMC1484078

[B36] Yavuz E. Turner J. Nowotny T. (2016). Genn: a code generation framework for accelerated brain simulations. Sci. Rep. 6, 1–14. 10.1038/srep18854 26740369 PMC4703976

[B37] York G. J. R. Osborne H. Sriya P. Astill S. de Kamps M. Chakrabarty S. (2022). The effect of limb position on a static knee extension task can be explained with a simple spinal cord circuit model. J. Neurophysiol. 127, 173–187. 10.1152/jn.00208.2021 34879209 PMC8802899

